# Integrative analysis of transcriptomic and epigenomic data reveals distinct patterns for developmental and housekeeping gene regulation

**DOI:** 10.1186/s12915-024-01869-2

**Published:** 2024-04-10

**Authors:** Irina Abnizova, Carine Stapel, Rene te Boekhorst, Jimmy Tsz Hang Lee, Martin Hemberg

**Affiliations:** 1 Epigenetics Programme, Babraham Institute, Cambridge, UK; 2 University of Hertfordshire, Hatfield, UK; 3 Wellcome Sanger Institute, Hinxton, UK; 4 The Gene Lay Institute of Immunology and Inflammation Brigham & Women’s Hospital and Harvard Medical School, Boston, USA

**Keywords:** Gene regulation programs, Differentially and similarly expressed genes, Developmental and housekeeping genes, Transcriptional architecture, Epigenomics, Pioneer TFs

## Abstract

**Background:**

Regulation of transcription is central to the emergence of new cell types during development, and it often involves activation of genes via proximal and distal regulatory regions. The activity of regulatory elements is determined by transcription factors (TFs) and epigenetic marks, but despite extensive mapping of such patterns, the extraction of regulatory principles remains challenging.

**Results:**

Here we study differentially and similarly expressed genes along with their associated epigenomic profiles, chromatin accessibility and DNA methylation, during lineage specification at gastrulation in mice. Comparison of the three lineages allows us to identify genomic and epigenomic features that distinguish the two classes of genes.

We show that differentially expressed genes are primarily regulated by distal elements, while similarly expressed genes are controlled by proximal housekeeping regulatory programs. Differentially expressed genes are relatively isolated within topologically associated domains, while similarly expressed genes tend to be located in gene clusters. Transcription of differentially expressed genes is associated with differentially open chromatin at distal elements including enhancers, while that of similarly expressed genes is associated with ubiquitously accessible chromatin at promoters.

**Conclusion:**

Based on these associations of (linearly) distal genes’ transcription start sites (TSSs) and putative enhancers for developmental genes, our findings allow us to link putative enhancers to their target promoters and to infer lineage-specific repertoires of putative driver transcription factors, within which we define subgroups of pioneers and co-operators.

**Supplementary Information:**

The online version contains supplementary material available at 10.1186/s12915-024-01869-2.

## Background

A central question in developmental biology is how different cell fates are obtained, with cell differentiation being driven in large parts through the control of gene expression [1]. Specific gene regulatory programs are required to control the timing and spatial location of gene expression [2–4]. In this regard, one can define two main types of genes of key importance. The first type is differentially expressed genes (DEGs), that is genes which are more strongly expressed in one lineage compared to another. Differential gene expression is crucial in development and many other complex biological processes [5–7]. The second type is genes whose expression is similar across cell types and developmental stages, and we refer to them as similarly expressed genes (SEGs). They are often called ‘housekeeping’ genes and are required for cell viability and basic maintenance [8–10]. Understanding genomic and epigenomic patterns of both housekeeping and developmental (or tissue-specific) genes is fundamental to understanding animal gene regulation [11–15].

Gene expression in eukaryotes is regulated in space and time by the interaction between promoters and distal cis-regulatory regions known as enhancers [16]. This process is influenced by the distance between promoters and enhancers, and promoter-enhancer specificity [3]. Here, specificity refers to the similarity of transcription factor binding motifs (TFBSs) found at the two loci. Moreover, it was suggested that promoter-enhancer sequence properties separate developmental vs housekeeping gene regulatory programs in *Drosophila* [17]. However, these studies did not consider epigenomic features that could contribute to possible distinct regulatory patterns of developmental and housekeeping genes.

Recent studies have begun to map in detail gene expression and epigenomic patterns during mouse gastrulation. Gastrulation is the emergence of the three primary germ layers, mesoderm, ectoderm, and endoderm, differentiation of which forms the basis for development of all organs in the adult body. One of the key insights that emerges from recent studies profiling DNA methylation, chromatin accessibility, and the transcriptome of individual cells is that enhancer marking is a lineage defining feature [15, 18].

Here we used datasets from [18] to define differentially and similarly expressed genes between the three germ layers, as well as their genomic distribution and epigenetic features, including in topologically associated domains (TADs), and epigenetic marking of their putative enhancers and promoters. The analysis provides new insights into the logic that underlies the regulation of developmental genes and housekeeping genes. By combining data from enriched TF binding motifs, expression levels and type of regulation (developmental or housekeeping/essential) of their corresponding genes we are then able to indicate putative pioneer factors and co-factors crucial for lineage differentiation.

## Results

### Genomic architecture of differentially and similarly expressed genes

**Fig. 1:**
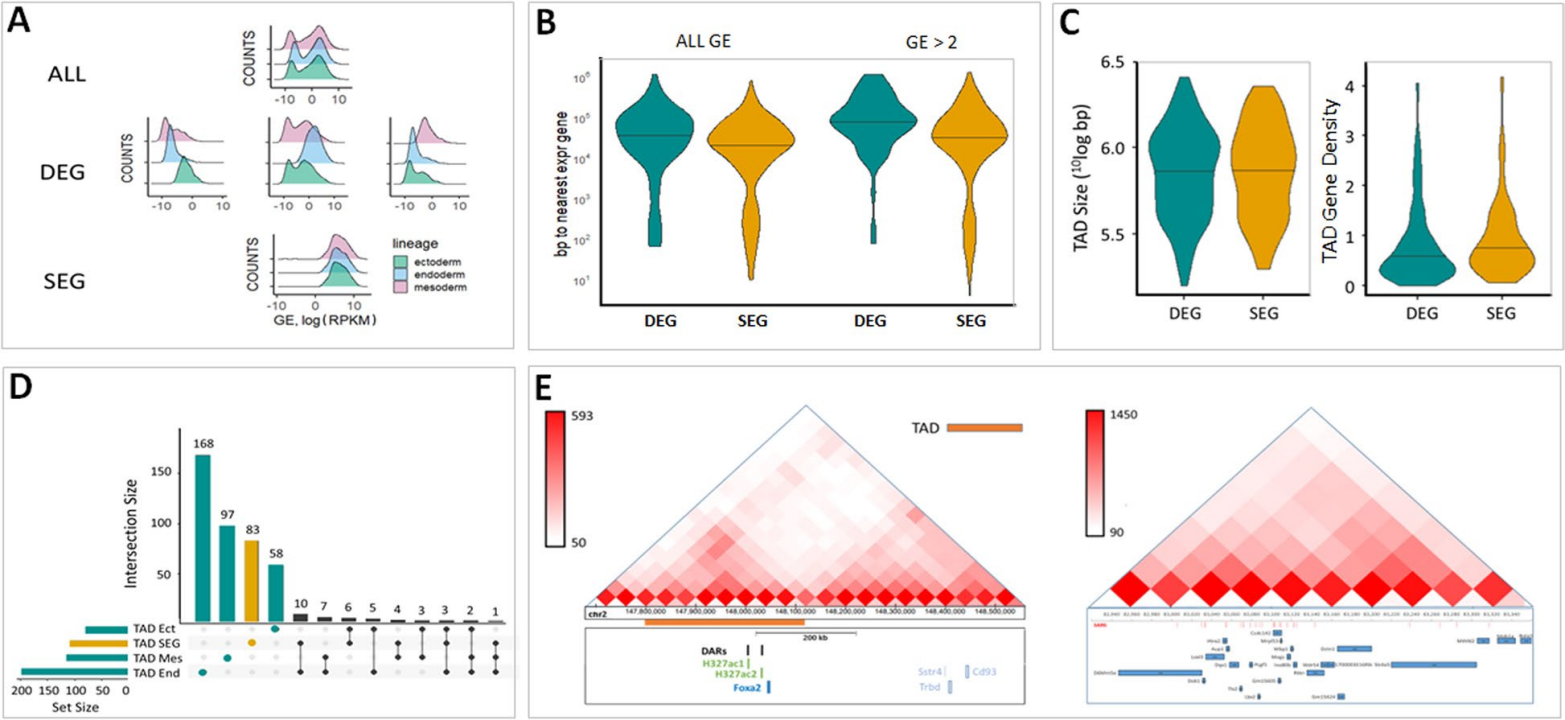
DEGs and SEGs, and corresponding TADs. **A** Gene expression (GE) distribution for each three lineages: for all genes (top), DEGs (middle) and SEGs (bottom). **B** Violin plots showing distances in base pairs to the nearest gene (TSS of gene to TSS of neighbouring gene) in a gene set depending on GE level: genes not depending on GE threshold (ALL GE, left), and genes with GE log (RPKM) > 2 (right). DEGs (blue, all three lineage-specific DEGs combined) are significantly further away from their neighbours than SEGs (orange, Mann-Whitney test, *p* < 0.01). **C** DEG- and SEG-only TADS do not differ in size (Mann-Whitney *p* = 0.79), but SEG-only TADS have a significantly higher gene density (computed and normalised in a 100-kB window) than DEG-only TADs (Mann-Whitney *p* = 0.021). **D** Upset plot showing content of TADs made up exclusively of genes expressed in just ectoderm, endoderm or mesoderm (DEGs, green), solely of similarly expressed genes (SEGs, yellow) and TADs whose content is an intersection of any two or three of the four sets. The dominance of coloured bars on the top left shows that the majority of TADs contain either DEGs or SEGs, with minimal intersections. **E** Hi-C interaction maps showing a typical DEG-only TAD (left) and a representative SEG-only TAD (right). The TAD on the left contains a single DEG (*Foxa2*), whereas the map on the right shows 16 SEGs sharing the same TAD. Genes are denoted by blue boxes, accessible chromatin by red boxes and known enhancers by green boxes. Long orange rectangle at the left plot shows the borders of the TAD, while the area under the interaction map on the right plot shows the whole SEG-containing TAD

During gastrulation, the cells of the embryo differentiate into three main lineages (ectoderm, endoderm, mesoderm), and differential gene expression between these has been extensively characterised [19–21]. We defined a set of differentially expressed genes (DEGs) for each of the three lineages at E7.5 using stringent criteria, based on significant differences in gene expression across the three lineages (Methods, Fig. 1A). Encouragingly, several well-known markers were found (Additional file 1: Table S1) amongst the 245 genes with exclusive high expression levels in ectoderm (e.g. Crabp2, Irx3, Sox2, Nav2), 771 genes with high expression levels in endoderm (e.g. Foxa2, Sox17) and 293 genes with high expression levels in mesoderm (e.g. Mesp1, Phida2, Lefty). Interestingly, there were more endoderm-specific genes, which reflected their relatively higher gene expression compared to the other lineages (Fig. 1A middle, Additional file 1: Fig. S1A). We also defined a set of similarly expressed genes (SEGs) which includes 1175 genes whose expression levels do not vary significantly across the three germ layers (Fig. 1A bottom).

We found that 61% of SEGs are known housekeeping genes (HKG) [13] (Fig. S2A), which is 3.9-fold more than expected by chance (permutation test, *p* < 0.0001). Manual inspection revealed several known housekeeping genes amongst the SEGs, e.g. *CTCF*, *Sf3b1* and *Eif2s3*. Accordingly, many DEG genes are known as developmental genes (Table 1). Gene ontology (GO) enrichment analysis confirmed the initial observation that DEGs are enriched for lineage-specific functions, while SEGs are enriched for basic cell maintenance terms (Table 1, Table S2). Interestingly, we also see a clear distinction in GO terms for molecular functions between DEGs and SEGs: SEGs are mostly involved in compound binding (organic cyclic, heterocyclic) and ribosome structure. Ectoderm and mesoderm DEGs are mainly involved in DNA-binding transcription activity, while endoderm DEGs are involved in transmembrane transporter activity and lipid binding. With respect to GO terms for cellular components, we found a significant prevalence of protein- containing complexes (while decreased proportion of anatomical entities) for SEGs than for DEGs (one sample *t*-test *p* = 0.0039, Fig. S2E).

**Table 1:**
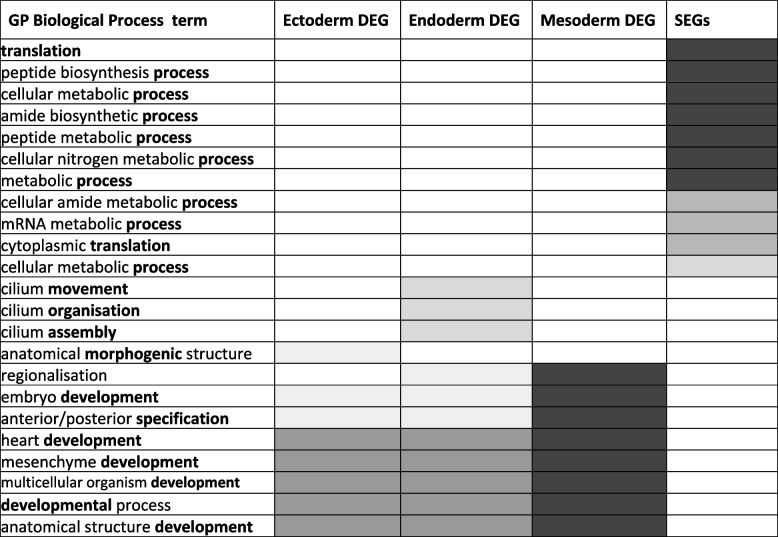
GO biological processes: DEGs vs SEGs

Where the shades of grey correspond to the following *p*-values:

Additionally, SEGs were similarly highly expressed across the developmental time points analysed (Fig. S2B), in agreement with HKG definition [8], in contrast to DEGs (Fig. S2C) which varied over time.

Another notable contrast between DEGs and SEGs is the CG content of their promoters. The majority of SEGs (72%, *p* < 8.079e−08 by hypergeometric/one tail exact Fisher test) have CG-rich promoters, while only 45% of DEGs have CG-rich promoters (Fig. S2D) consistent with previous reports that housekeeping genes have CG-rich promoters [22]. It has also been reported that genes with CG-rich promoters tend to be more highly expressed [23], and our analysis reveals that SEGs tend to have significantly higher expression levels than DEGs (Mann-Whitney test, *p* = 2.2e−16, Fig. 1A bottom, Additional file 1: Fig. S1B).

It has been shown that the location of genes throughout the genome is non-uniform, with both larger clusters (gene-dense regions) and deserts (gene-poor regions) more frequent than expected by chance [24]. We asked if DEGs and SEGs are more or less likely to be part of gene clusters by calculating their distances to the nearest expressed gene. We found that DEGs are located further away from other genes compared to SEGs, regardless of the threshold used for deciding if a gene is expressed (Fig. 1B, Fig. S3). This shows that DEGs are more isolated and more likely to be found in relatively gene-poor regions, while SEGs are more likely to be found in relatively gene-dense regions. Since SEGs on average have higher expression levels than DEGs, we also selected a subset of genes from each group that were matched by expression levels. This additional control confirmed that the density of SEGs and their high CG promoter content were not a consequence of high expression levels (Fig. S3).

Our finding is consistent with earlier reports suggesting that some key developmental genes, such as *Hoxd* or *Myc*, are flanked by gene-poor regions [24–26], as well as studies showing that some HKGs are clustered [27, 28]. To the best of our knowledge, we present the first systematic study to address the question of genomic location difference between developmental and HKG genes in a mammalian genome.

### Arrangement of DEGs and SEGs within topologically associated domains (TADs)

Although chromosomes are linear, they are folded in the cell nucleus, resulting in a characteristic 3D organisation which has been shown to be important for understanding gene regulation. In particular, topologically associated domains (TADs) are defined as regions of increased internal chromatin contacts [29, 30] which impact target gene regulation by enhancers and other cis-regulatory elements located within a TAD [31]. TADs are largely conserved throughout the lifespan of mammalian organisms and are established as early as the inner cell mass stage (which precedes gastrulation by 3 days) in mice [15, 32]. Since the genes found inside a TAD tend to share regulatory interactions we investigated the location of DEGs and SEGs relative to TADs [29, 33].

We found that DEG-containing TADs have a lower gene density than SEG-containing TADs (median values are 0.65 and 0.8 genes per 100 kB, *p*-value = 0.012 Mann-Whitney test, Fig. 1C). We also found that fewer DEGs and SEGs are located in the same TAD than expected by chance (*p* < 0.001 permutation test, Fig. 1D). Thus, we conclude that DEGs and SEGs are mostly found in separate regulatory domains and that DEGs are less likely to share regulatory interactions with other genes (Fig. 1E, Additional file 1: Fig. S4).

### Relationship of DEGs and SEGs to chromatin accessibility

Chromatin structure plays a key role in regulating gene expression by determining DNA accessibility to allow transcription factors, RNAPII, and Mediator complexes to bind [34, 35]. Consequently, we hypothesised that differences in lineage-restricted expression found in DEGs might be reflected in the arrangement of accessible chromatin.

We developed an unbiased genome-wide method to identify differentially and similarly accessible regions for pseudo-bulked NMT-seq data (Methods). We defined a set of differentially accessible regions (DARs) for each of the three lineages at E7.5, and using stringent criteria we obtained regions exclusive to each lineage: 33,005 regions highly accessible in ectoderm, 73,442 regions highly accessible in endoderm and 31,543 regions highly accessible in mesoderm. The larger number of endoderm DARs reflects the larger number of endoderm DEGs. We also defined a set of similarly accessible regions (SARs) consisting of 169,088 regions with similar chromatin accessibility levels across all three germ layers.

**Fig. 2:**
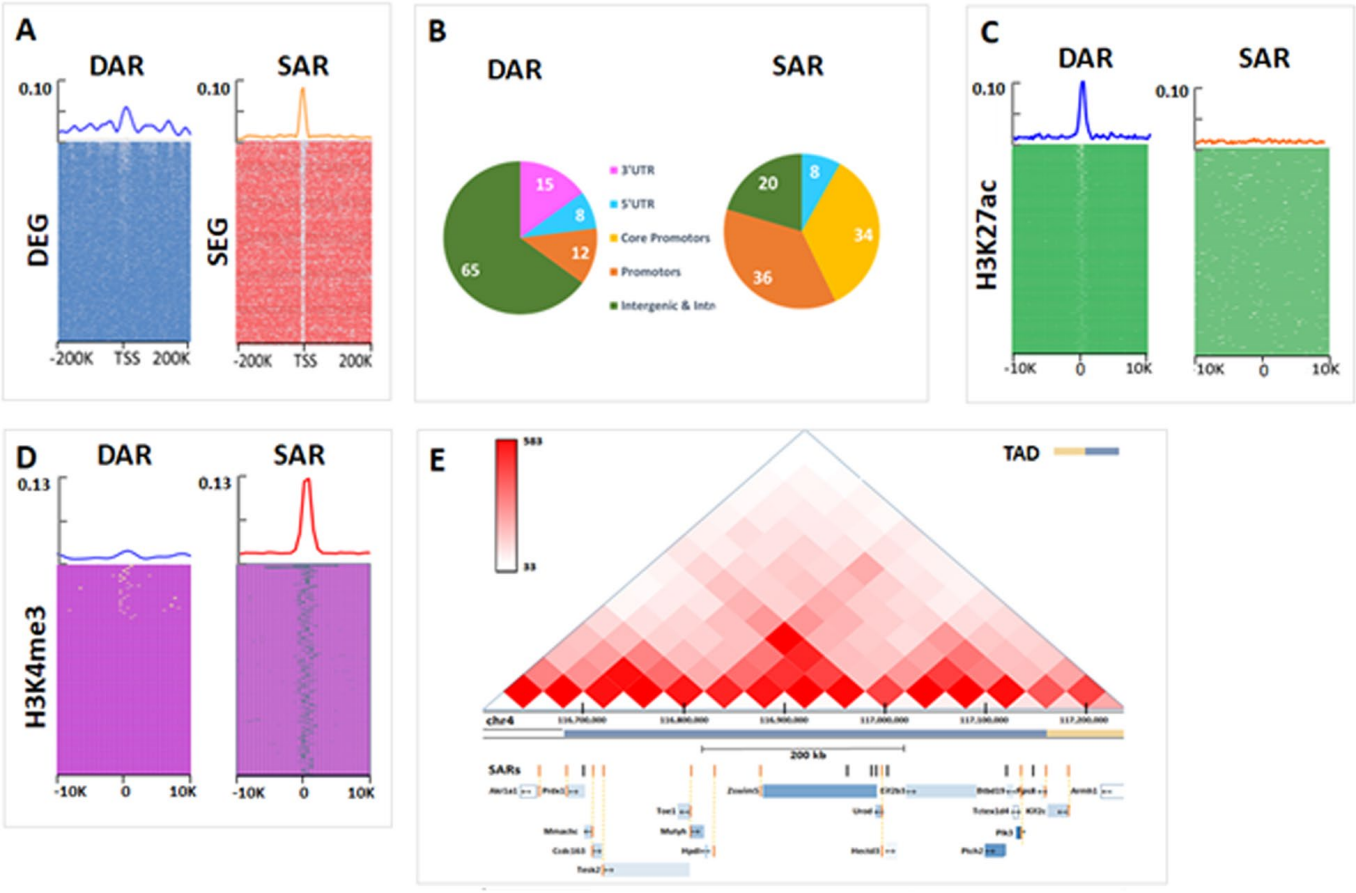
Differential and similar chromatin-accessible regions (DARs and SARs) properties. **A** Distribution of DARs (blue, left) and SARs (red, right) relative to the TSS of DEGs (blue heatmap) and SEGs (red heatmap) in 5-kB bins. Heat maps show occurrences of DARs/SARs around each gene TSS. **B** Pie chart of genome-wide distribution of DARs and SARs. **C** Clustering of matched DARs (blue line, left heat map) around lineage-specific H3K27ac enhancers (green heatmaps), and SARs (red line, right heatmap) around H3K27ac for comparison. **D** (left) Distribution of DARs (blue line) around H3K4me3 peaks (violet heatmap); (right) distribution of SARs (red line) around H3K4me3 peaks (violet heatmaps). **E** An example of SEG-populated TAD and SARs within it, with SARs aligned with SEG gene’s promoters for 15 of the 16 promoters

Both DARs and SARs are more abundant in the vicinity of actively transcribed genes (Fig. 2A), consistent with the hypothesis that they serve as putative regulatory elements. However, we found a pronounced difference between the spatial distribution of DARs and SARs in relation to the TSSs of DEGs and SEGs respectively (Fig. 2A, left vs right). DARs are broadly distributed in a large region around the TSSs of DEGs (Fig. 2A, left). Moreover, most DARs are intergenic or intronic (Fig. 2B), and their CG content is not different from the genome-wide average (Additional file 1: Fig. S5B). By contrast, SARs are predominantly found within 2 kB of the promoters of SEGs (Fig. 2A right), and they often have elevated CG content (Additional file 1: Fig. S5B), presumably due to the proximity of the CG-rich promoters. Interestingly, a similar pattern for the distribution of enhancers associated with developmental and housekeeping genes was reported previously in *Drosophila* [17].

Another indication of the potential regulatory role of DARs comes from the analysis of ChIP-seq derived H3K27ac lineage-specific active enhancer marks (annotation from [18], data from [36]) also profiled at E7.5 in the three lineages. Our results show a high degree of overlap between DARs and lineage-specific H3K27ac marks (Fig. 2C), indicating an enrichment of DARs around active enhancers. However, there is no enrichment of SARs around H3K27ac (Fig. 2C right).

We also aligned the sets of DARs and SARs to early mouse H3K4me3 histone marks [18] to investigate the distance to active promoters. This revealed (Fig. 2D) that SARs are intensively clustered around H3K4me3 centres, which is expected. In contrast, < 1% of DARs are found near H3K4me3 peaks, supporting our conclusion that DARs are likely distal regulators.

An illustration of the proximity of SARs and SEGs is shown in Fig. 2E. In this SEG-dense TAD there are many known HK genes, with 15 out of 16 genes having SARs (vertical yellow lines) within their 5’ promoter regions and 13 out of 16 genes having a H3K4me3 peak. Taken together, these findings indicate that DARs and SARs are likely to participate in different regulatory programs. DARs appear to contribute to long-range regulation of developmental genes, while SARs are likely to be involved in short-range control of housekeeping genes.

### Linking putative distal regulatory elements to target gene promoters

**Fig. 3:**
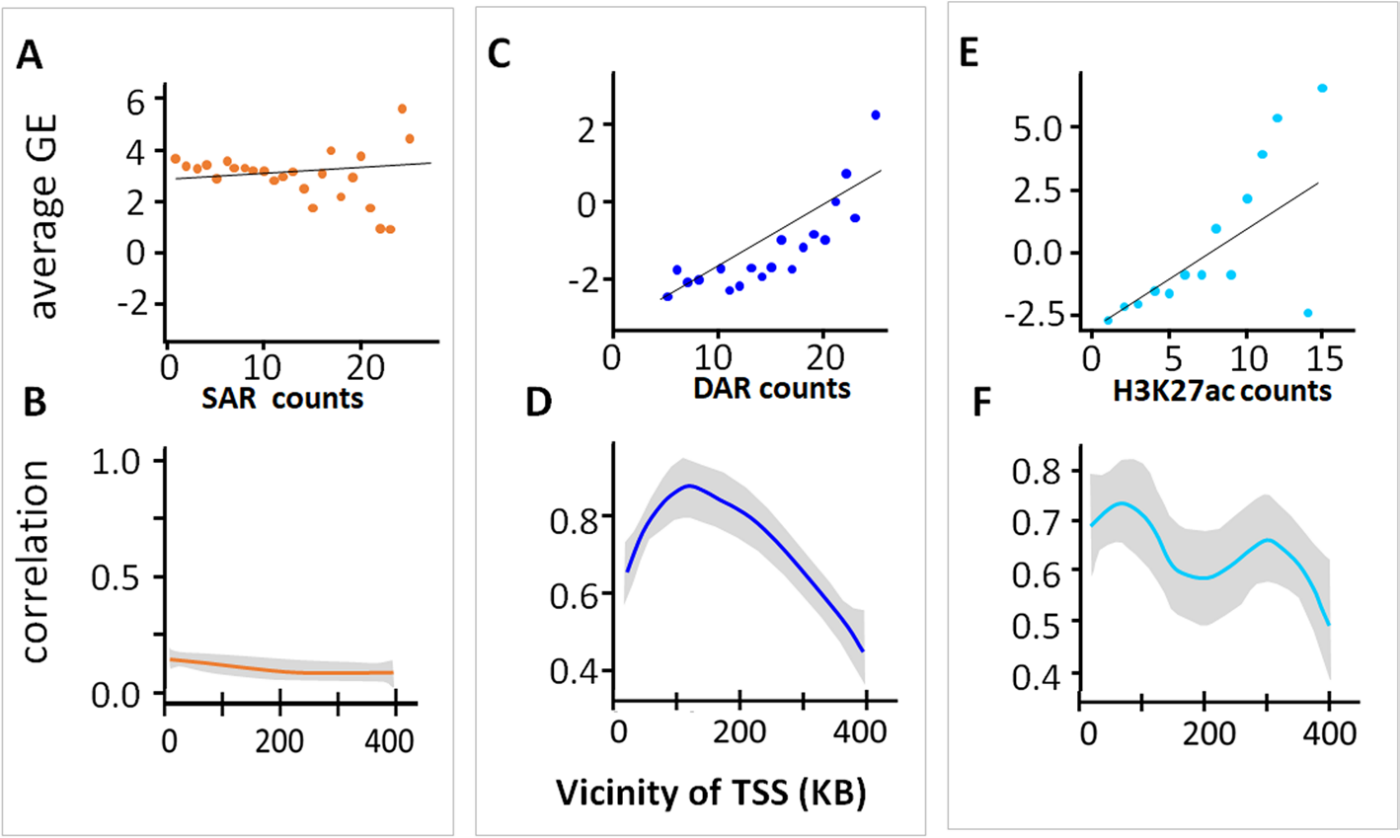
Long-range correlation of gene expression and frequency of chromatin-accessible regions. **A** Low correlation of SAR frequency and average gene expression of SEG sets in a 80-kB TSS vicinity of SEGs (*R* = 0.08, *p*-value >0.05). **B** SARs and SEGs are not correlated across 400 kB, *R* < 0.15, *p*-value>0.05 over 400 kB. **C** Correlation of DARs frequency (Methods) and average gene expression of corresponding DEG gene sets in a 80-kB TSS vicinity of DEG’s TSS, *R* > 0.7, *p*  <0.05. **D** Zones of ‘influence’ (positive correlation of accessibility and gene expression) for DARs - DEGs, *R* > 0.7, *p* < 0.05. **E** Correlation of H3K27ac frequency (Methods) and average gene expression of corresponding DEG gene sets in a 80-kB TSS vicinity of DEGs TSS, *R* > 0.7, *p* < 0.05. **F** Zones of ‘influence’ (positive correlation of accessibility and gene expression) for H3K27ac - DEGs, *R* > 0.7, *p* < 0.05 for maximal correlation around 100 kB

Although many distal accessible loci represent enhancers that may regulate gene expression levels of their target genes [37], enhancers are a heterogenous class of genomic elements, and thus we asked whether DARs and SARs impacted gene expression in the same or in different ways. We assumed that an enhancer in general will have a positive impact on gene expression of its nearby genes [38, 39], and we developed an open chromatin abundance coefficient (CAC) to quantify the association between chromatin accessibility and gene expression. For a given set of matched genes and putative enhancers (e.g. ectoderm DEGs and ectoderm DARs), the number of accessible regions in a fixed vicinity of each TSS of the gene set is computed and divided by the number of expressed genes in the same region (Methods). The CAC is then computed as the Pearson correlation coefficient between average gene expression levels and the normalised frequency of accessible regions across all genes in the set, as in Fig. 3 top panel. A high CAC value means strong positive association between average gene expression levels and the normalised frequency of open chromatin regions around the corresponding genes. We computed the CAC separately for each lineage and for the three sets of DEGs and SEGs, for a range of regions around TSS (Fig. 3 bottom panel). We did not find an association between SARs and SEGs (*R* = 0.08, *p*-value > 0.05, Fig. 3A, *R* ≤ 0.15 across 400 kB Fig. 3B, *p* > 0.05). The absence of such an association can be explained by our finding that SEGs are close to other expressed genes and are likely to share regulatory regions, or regulate each other through their promoters or gene bodies [39]. This low correlation over a big range of distances is consistent with the notion that SARs primarily function as proximal regulatory elements.

By contrast, we found a strong lineage-specific positive association between average expression of DEGs and the frequency of DARs located at distances between 50 and 400 kB of their TSS (Fig. 3C, D). The CAC score first increased within short distances of the TSS, with a global maximum at ~100 kB of the TSS (*R* > 0.7, *p*-value < 0.001). Then it decreases monotonically with distance up to 400 kB (Fig. 3D). Interestingly, this range roughly corresponds to the sizes of TADs [40]. We also investigated lineage-specific active enhancer marks (H3K27ac) identified in a previous study [36], and unsurprisingly given their overlap with DARs they also have high CAC scores (Fig. 3E, F). In contrast, an epigenomic mark for active promoters, H3K4me3 [18], did not correlate with GE (data not shown) and showed very small overlap with DARs.

Based on the maximal association in the vicinity of the TSS, we developed an algorithm for linking DEGs and matched lineage-specific DARs/putative enhancers (Methods). The inputs of the algorithm are a set of genes and putative regulatory regions for each lineage. The output is a catalogue of 960 DARs for ectoderm, 3756 for endoderm, and 1352 for mesoderm which are putative enhancers linked to genes. Encouragingly, some well-known combinations of developmental genes and their enhancers (e.g. *Shh*, [41], (Fig. S4C), *Cxcl12*, *Mesp2* (Fig. S4D) [18, 41]) were captured by our method. However, the majority of connections have not been reported in the literature before and hence represent novel candidate regulatory regions for these genes. This procedure for linking enhancers to promoters is not required for SARs since the majority of them are located within the promoter of a SEG. We conclude that the CAC score can be used to link DARs and DEGs, while no such association is required for SEGs and SARs.

### Identification of differentially methylated and similarly methylated regions

DNA methylation affects gene expression in several ways, including by recruiting proteins involved in gene repression or by inhibiting the binding of transcription factors to DNA [42–44]. We defined a set of differentially hypomethylated methylated regions (DhMR, a small ‘h’ in DhMRs denotes low methylation level). We did it for each of the three lineages at E7.5 using stringent criteria (Methods). Since DNA methylation regions are typically longer than nucleosome-depleted chromatin-accessible regions [42, 45, 46], we computed DhMRs/ShMRs in a larger window (500 bp) than DARs/SARs (150 bp). We obtained 1759 hypomethylated regions in the ectoderm, 12,669 hypomethylated regions in the endoderm and 2975 hypomethylated regions in mesoderm. We also defined a set of similarly hypomethylated regions (ShMRs) consisting of 17,603 regions with similarly low DNA methylation levels across all three germ layers.

**Fig. 4:**
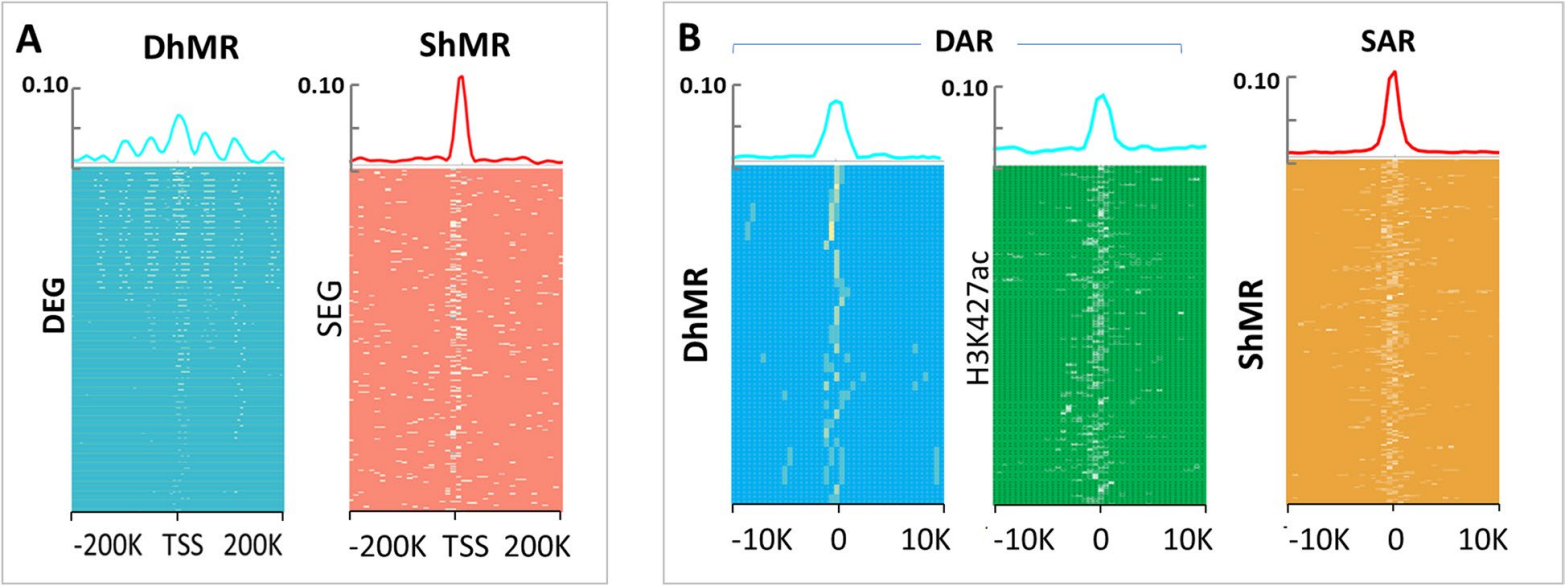
Differentially and similarly DNA hypomethylated regions. **A** DhMRs relative to TSS DEGs (left, light blue heatmap) and ShMRs relative to TSS of SEGs (right, red heatmap). **B** DhMRs are clustered around DARs (left, light blue heatmaps); DhMRs are clustered around H3K27ac (middle, green heatmaps); ShMRs are clustered around SARs (right, orange heatmaps)

The lower number of DhMRs/ShMRs compared to DARs/SARs is most likely due to the fact that there were ~10 times fewer reads for DNA methylation, a property of the scNMT-seq technology [47]. However, 18% of DARs are clustered around a DhMR, and 66% of SARs are clustered around a ShMR. These overlaps are significantly greater than expected by chance (permutation test, *p* < 0.001). Reassuringly, we observed that similar to SARs, ShMRs are closely clustered around TSSs of SEG (Fig. 4A right), while DhMRs were broadly distributed around DEGs (Fig. 4A left), similarly to DARs (Fig. 2A). Moreover, DhMRs overlap with both DARs and active enhancer marks (H3K27ac), Fig. 4B left and middle, while ShMRs overlap with SARs, Fig. 4B right.

### Transcription factor binding sites in DEGs and SEGs: enhancer-promoter difference

Finally, we wanted to understand if TF binding repertoires are different for developmental (DEGs) and housekeeping (SEGs) regulatory programs. As reviewed in [3], developmental gene promoter-enhancer activation is likely regulated via a dynamic hub of a ‘transcription factory’: a collection of multiple TFs, co-factors, RNAPII and mediator. The process of activation is influenced by promoter specificity, distance and possibly pre-configuration [3].

However, less is known about how promoters of housekeeping genes are activated [13]. One hypothesis is that different core promoters possess an internal specificity, manifested by similarity in sequence composition, for certain enhancers [48–50]. The existence of such ‘sequence-encoded enhancer-promoter specificity’ was confirmed by [51] for the *Drosophila* genome, where the authors studied TF binding site motif repertoires contributing to developmental and housekeeping promoter-enhancer specificity.

From our analysis of the distribution of open and hypomethylated chromatin around TSSs of DEGs and SEGs, we identified two general sets of ‘regulatory neighbourhoods’ (Methods): (i) a developmental neighbourhood, which links distal differentially accessible and hypomethylated chromatin (DARs and DhMRs) with DEGs (Additional file 1: Fig. S7A top), and (ii) a housekeeping neighbourhood, consisting of SARs and ShMRs proximal to SEGs (Additional file 1: Fig. S7A bottom). Using these notations, we can examine if there is TF motif-based similarity within and between developmental and housekeeping regulatory neighbourhoods, i.e. if DEG enhancers are more similar to DEG promoters than to SEG promoters.

We developed a method to measure overall similarity between motif repertoire (Methods) and we used a permutation test to assess its significance, and we applied it to infer promoter-enhancer specificity for the two neighbourhoods defined above (Methods, Additional file 1: Fig. S6). We found that based on the motif sets’ ranked similarity scores, DEG core promoters are significantly more similar between each other, compared to SEG core promoters (*t*-test, *p* = 0.032) (Table S3.1). We also found that based on ranked motif scores, DARs are significantly different from SARs (*t*-test, *p* = 0.012), see Table S3.2. Finally, we found significant motif-based differences between corresponding DARs and core promoters of DEGs, as well as SARs and core promoters of SEGs (Table S3.3 detailed, ANOVA *p* = 0.056). We identified two sets of motifs contributing to DEG vs SEG promoter-enhancer specificity and communalities.

Both DARs and DEG core promoters contained TATA-like boxes, Rfx, Wt1 families, and DPE-like motifs (STAT family, containing STTC pattern). Interestingly, some of the common motifs, such as MAZ, Zic2, Zic3, contained the ‘GAGG’, ‘CAGA’ - patterns, which are similar to ‘GAGA’ of Trl in *Drosophila*, in line with what was found in [51].

By contrast, SARs and core promoters of SEGs were enriched for ELF families, GABPA, YY1, NRF, Creb and TCT-like motifs. These motifs are known [4] to control expression of housekeeping genes, and they are typically associated with open chromatin [52, 53] and high GC-content promoters [54]. Taken together, our findings support the hypothesis of a ‘sequence-encoded enhancer-promoter specificity’ for the mouse genome, similar to what was reported by [51] for Drosophila.

### Analysis of motif features contributing to sequence-encoded enhancer-promoter specificity

We studied the motifs specific to SAR-SEG promoters, defined as the intersection of high-ranked motifs and motifs specific to DAR-DEG promoters, defined as the union of all three DAR-DEG high-ranked motif intersections. We wanted to learn if any sequence features, such as nucleotide content, complexity and motif length, were different between these two sets of motifs. Interestingly, the complexity, as defined by sequence di-nucleotide entropy, of SAR-SEG promoter motifs is significantly lower (Mann–Whitney, *p* = 2.5e−05, Additional file 1: Fig. S6A) than the DAR-DEG specific motifs. We also found elevated A-content of DAR-DEG specific motifs (Mann-Whitney, *p* = 0.0031, Additional file 1: Fig. S6B), probably because of the 0-on bdcp Purine GAAA-pattern in putative developmental enhancers and DEG core promoters (MAZ, ZIc1, Zic2 contain this pattern). Similarly, Zabidi and Stark [51] found GAGA-pattern (also Purine) activating developmental genes distally in the fruit fly. Finally, we found that DAR-DEG-specific motifs are around 5 bp shorter (Mann-Whitney, *p* = 0.00073, Additional file 1: Fig. S6C) than SAR-SEG specific.

### Inferring lineage-specific driver TFs from motifs in putative enhancers

**Fig. 5:**
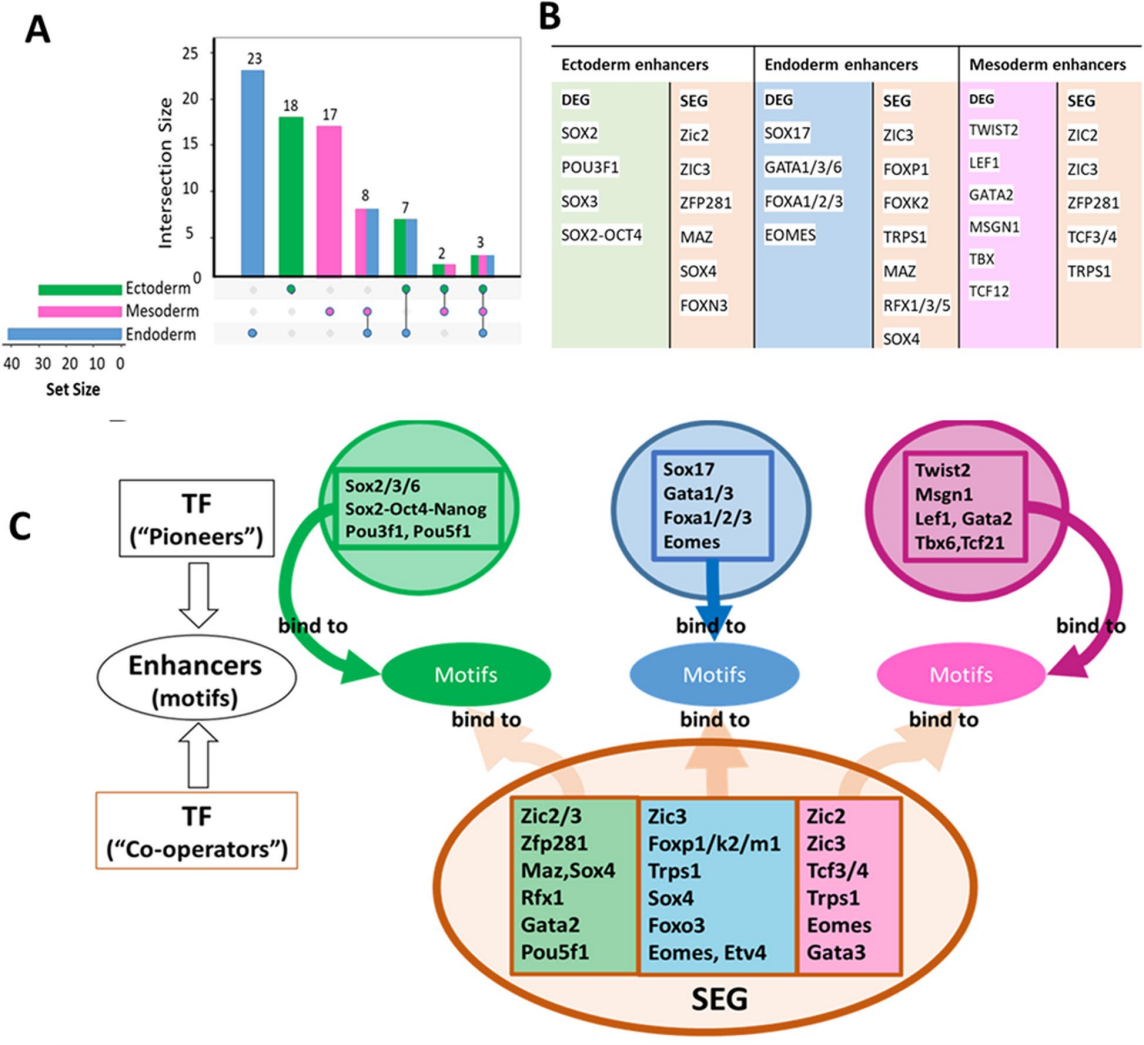
Inferring lineage-specific sets of driver TFs. **A** The upset plot for significantly enriched motifs (*p* < 0.001) within lineage-specific putative enhancers. Colours are for lineage-specific lists: green for ectoderm, blue for endoderm, pink for mesoderm. **B** TFs binding to lineage-specific enhancers: their TFBS motifs are most enriched (*p* < 0.001) within DEG’s lineage-specific putative enhancers, filtered by GE > 0 of their corresponding genes. The genes are expressed in their lineage and the corresponding motif is enriched in the lineage-specific regulatory regions. **C** Green, blue, pink coloured boxes within circles (DEGs) at the top contain pioneer driver DEG-produced TFs. The orange-rimmed boxes contain lineage-specific binding TFs (correspondingly coloured background), presumably cooperative TFs; their genes are expressed in all three lineages (SEGs). Coloured ovals denote distal putative enhancers with cis-regulatory motifs for corresponding TFs

We next examined TF motifs that distinguished lineages. It is assumed that motif-specific distinction within enhancers likely drives lineage differentiation [55, 56]. We found that TF binding sequence motifs in DARs are mainly distinct between lineages (Fig. 5A). We show the most enriched lineage-specific TF motif repertoires in Fig. 5B, coloured by a lineage whose genes are exclusively expressed.

Consistent with expectations, many of the TFs whose motifs are enriched (Fig. 5B) are known to be important in lineage specification and pattern formation, such as Pou3f1 [11], Sox2 and Sox3 [57–59] in ectoderm; Sox17, Foxa1 [60, 61], Gata3 [62], Gata1 [63], Gata6 [64], Eomes [65], Fox-factors [60, 66–68] in endoderm, Msgn1 [69], Twist2 [70], Lef1 [71], and Tbx1 [72], Tcf12 [73, 74] in mesoderm. Interestingly, within enriched TF binding sequence motifs (Fig. 5C) around 40% had at least one corresponding TF gene expressed as DEGs, and they are also known to be pioneer factors (Table S4) [75, 76] and important in lineage specification (Fig. 5C green, blue, purple lists). The remaining 60% of enriched TF binding sequence motifs had their corresponding TF genes expressed in all three lineages (SEGs, Fig. 5C orange sub-lists) and the TFs were not classed as pioneer factors (Table S4). This finding supports the hypotheses: (i) that pioneering factors regulate developmental networks [77] and (ii) developmental context also influences pioneer-factor binding and activity [78]. In contrast, one does not need pioneering properties to bind on open chromatin around SEGs/HKGs to control them.

## Discussion

We have characterised the genomic and epigenomic properties of similarly and differentially expressed genes in mouse gastrulation. By combining datasets from multiple modalities, we have exposed the differences between two key processes during embryonic development. In principle, a similar approach can be applied to other scenarios, in which more general biological processes coincide with more specific ones, such as the immune response and properties of lymphoid cells, or to infer cell-type-specific sets of TFs.

One important implication of our findings includes the possibility to link developmental enhancers/DARs/DhMRs to their target DEGs. Linking enhancers to their target promoters remains a challenging problem [79–82]. To the best of our knowledge, the most successful approach so far was to link promoters to enhancers based on 100 K distance proximity of chromatin-accessible regions [83–87], without considering possible interference of neighbouring expressed genes. Our approach, which takes into account distance to the nearest transcribed gene, chromatin accessibility, and TAD borders, could allow for the detection of more precise promoter-enhancer links for developmental genes. Our approach also allows to characterise enhancer-promoter specificity, which separates developmental from housekeeping regulatory programs for mouse gastrulation data. Importantly, our algorithm does not require chromatin contact information, making it more widely applicable. Although previous studies of *Drosophila melanogaster* development and macrophage differentiation have indicated that regulation of housekeeping genes is distinct from cell-type-specific genes [17, 88–90], these studies did not address the contribution of genome architecture and epigenetic patterns.

Interestingly, SARs and ShMRs are more often co-localised compared to DARs and DhMR. This difference might be due to the divergent biological functions of DARs and SARs and their location relative to the TSS of corresponding DEGs and SEGs. Most SEGs are highly expressed and their promoters are thus hypomethylated [91]. In fact, SARs frequently overlap both active promoters and ShMRs.

**Fig. 6:**
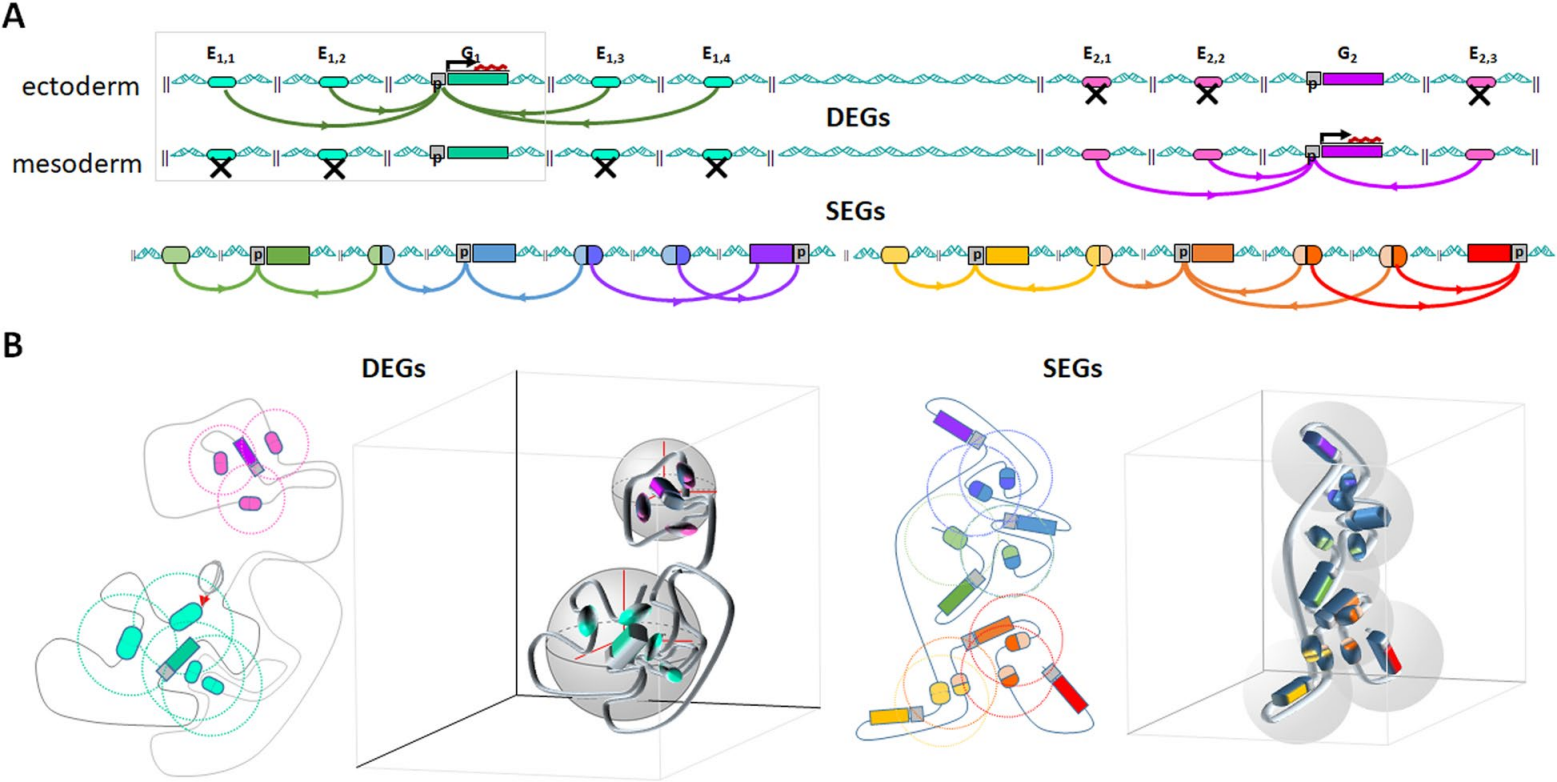
Schematic diagram illustrating relative isolation of DEGs compared to the clustering of SEGs: **A** as a linear sequences; **B** as a two dimensional loops and three dimensional folding: Lineage-specific promoter-enhancer activation is indicated by colours (ectoderm—green, mesoderm—pink). Ovals (ectoderm—green, mesoderm—magenta) denote distal regulatory regions, crossed ovals are closed (chromatin inaccessible) enhancers. Genes are coloured elongated rectangles, promoter regions / TSS are grey squares. The grey-coloured circles with an oval at the centre represent a presumed radius of activation of a regulatory element and include the promoter region / TSS of its target gene(s). They correspond to the connecting arcs in the sequential representation (**A**) but demonstrate that linearly far away may be nearby in 2D

Here we confirm and refine the notion of ‘insulated neighbourhood’ [49], which states that TADs are stable chromosomal structural regulatory units. It was further observed that a constant stable structure mostly holds for TADs harbouring HKGs [92]. We therefore introduce a regulatory neighbourhood notion, which reflects more upon functional interactions within TADs, rather than only structural features [49]. A possible spatial model of regulatory neighbourhoods is shown in Fig. 6. It illustrates our observations about architectural features of DEGs vs SEGs. For DEGs, the regulatory neighbourhoods are clearly separated from one another, and likely to be more dynamic together with their sub-TADs’ borders [3], whereas for SEGs they form a ‘foamy’ structure of intersecting bubbles at a higher, probably more global, stable and less specific, level of regulatory organisation.

Additionally, we identified putative lineage-driving TFs (Fig. 5B left sub-lists) which coincide with known pioneer factors. Indeed, the separation of enriched binding motifs according to the origin of their cognate TF’s gene expression strongly suggests that DEG-expressed TFs are often pioneers. Therefore, these pioneer TFs are likely to drive cell fate transitions [78, 93] and lineage differentiation. We interpret our findings as the following process of promoter-enhancer activation (Additional file 1: Fig. S8): pioneer TFs (DEG-originated lists Fig. 5B, green, blue and magenta backgrounds) bind closed chromatin of putative enhancers, and later recruit non-pioneer factors (SEG-originated lists Fig. 5B, orange backgrounds) to establish a nucleosome-free region. Together these dynamic clusters, which include not only TFs but also co-factors, co-activators (e.g. p300 and CRB), RNAPII and mediators, connect to the promoter. To the best of our knowledge, this association between DEGs and pioneer factors has not been previously reported, and it has important implications for understanding how the gene expression programs specific to each lineage is established.

Although we have identified important characteristics of two key regulatory programs, many questions remain. Undoubtedly, not all developmental genes are DEGs during gastrulation. Some developmental genes can be DEGs at earlier stages of embryogenesis [15, 32], e.g. important pluripotency genes Sox2, Oct4 and Nav1 are already highly expressed at E4.5. As we have only focused on a subset of genes at one stage of development, it is likely that other regulatory principles could also play a role. Another challenge stems from the fact that there might be multiple functions for the same gene [94, 95] and it remains unclear how different expression patterns are supported. Finally, for future research, it would be desirable to measure TF binding directly, not only inferring it via motif enrichment.

One might argue that in contrast to being isolated as we suggest here, there is a well-known canonical example of clustered developmental genes, namely the *HOX* genes [96]. However, at E7.5 day these genes were not yet expressed, and hence it is not obvious that they should be considered a counterexample to our model. Nevertheless, *HOX* genes are known to be spatially and temporally co-linear [97] and their chromatin landscape is known to be dynamic in time [98]. It is also known [99] that they are flanked by gene deserts and activated by different enhancers. Therefore, it is very likely that although they are spatially close to each other, they are transcriptionally far away from any other transcribed genes at any fixed time point, being activated sequentially while being transcriptionally isolated.

## Conclusions

We have shown that genes at the extreme ends of the similarity spectrum, DEGs and SEGs, differ from each other with respect to their distance to the nearest expressed neighbouring gene transcription start site, local chromatin accessibility and DNA methylation. At developmental time E7.5, DEGs are isolated within the genome and are regulated by distal putative enhancers (lineage-specific hypomethylated and chromatin-accessible regions and H3K27ac). In contrast, SEGs are more clustered and regulated by proximal chromatin-accessible and hypomethylated (within all three tissues) regulatory regions. As DEGs correspond to developmental genes and SEGs to housekeeping genes, we conclude that gene regulatory programs for developmental and housekeeping are distinguished by the predominantly distal vs proximal promoter-enhancer interaction.

Based on the separation above (corresponding epigenomic regulatory neighbourhoods), we infer putative lineage-driving TFs and their co-factors based on TFBS enrichments within putative distal enhancers. Interestingly, developmental programs produce pioneer TFs. Our results therefore provide new insights into the interaction of spatio-temporal genomic and epigenomic layers in the context of two contrasting regulatory programs governing developmental and housekeeping genes.

## Methods

### Definition of DEGs and SEGs

To identify the differentially expressed genes (DEGs) in each of the three lineages, and one set of similarly expressed genes (SEGs), the strategies as outlined below were used.

To identify DEGs:

- A gene is differentially expressed between two lineages if the difference in its expression is >3-fold with a *p*-value < 0.01 as determined using the MAD-score estimate [100]. We require that a gene is differentially expressed when compared to both the other lineages.
- To avoid multiple pairwise comparisons with subjective thresholds, we adopted the Berger-Parker Dominance index [101]. It is widely used in ecology to measure variability of species and it is also used by Illumina for base call quality identification (GATK documentation [102]). For any gene *g* with gene expression GE = (*GE1,GE2,GE3)* (*GEi* is gene expression in each lineage here), the Dominance index between values *GE1*, *GE2*, and *GE3* is the ratio of *max(GEi)* to the sum of all three values:
  $$Dom=max(GEi)/\sum GEi$$

A well-established threshold *Dom* = *0.6* [102–104] indicating a strong dominance of one lineage was applied to define DEGs.

(c) Unsupervised clustering using seq_monk [94, 105] with default parameters for DESeq2 [106] application.

The SEGs across all three lineages were obtained by the following strategies:

- Minimization of Dominance index across three lineage’s values, as low as *Dom* = *0.34*. Note that completely even distribution is indicated by *Dom* = *0.33*, and we use *0.34* as threshold to indicate almost even distribution. Setting this Dom threshold, we aimed to obtain an amount of SEGs comparable with the amount of DEGs. Therefore, a threshold *Dom* = *0.34* indicating an almost even dominance of all three lineages for a gene was applied to define SEGs.
- Minimization of MAD-score across three lineage’s values.

Each DEG-identification strategy resulted in three sets of DEG genes (ectoderm, endoderm and mesoderm uniquely expressed genes). The sets were of comparable sizes (around 700 genes for ectoderm and mesoderm, and around 1000 genes for endoderm). The lists of genes for each strategy were highly overlapping, their Jaccard similarity was around 0.9. Moreover, all further analysis results were valid for each strategy’s set of genes. Sets of known marker genes for each lineage [47, 107] were used to optimise and calibrate our chosen DEG sets. Based on this criterion, the Dominance index-derived DEG sets were chosen to capture the maximum number of known marker genes for gene sets of roughly the same size. We used the Dominance index distribution to determine the high dominance value, *D*_*high*_ = *0.6* [102], which we picked as threshold for highly dominant (in one lineage) genes, DEGs. For SEG identification, we collected all genes with almost minimal Dominance equal *D*_*low*_ = *0.34* (equally valued across the three lineages) here.

To determine our threshold significance, we ran a permutation test. For SEGs, we randomly and uniformly picked one gene expression level within each lineage (across all expressed genes at least in one layer) 1000 times, creating 1000 random three-tuples, which are our simulated genes’ GE. Then we compute Dom for each simulated gene.

The *p*-value of our hypothesis H0 for SEGs (H0: a value *Dom* = *0.34* can be obtained by chance) is estimated as the proportion of permutations that give a *Dom* value ≤ 0.34. Here, the null hypothesis can be rejected with a *p*-value of 0.001 since none of the permutation gives a Dom value ≤ 0.34.

For DEG’s threshold *Dom* = *0.6*, we conducted a permutation test similarly, but we sampled from a set of genes expressed in one of the layers (because our DEGs should be expressed in a corresponding layer). For example, we sample from a set of genes with GE > 2 in ectoderm for the ectoderm layer set. In this case our null hypothesis is that Dom = 0.6 for ectoderm layer genes can be reached by chance for any permuted gene with GE_ect > GE_mes and GE_ect > GE_end (according to DEG definition). In contrast, we have shown that it is very unlikely to get value Dom = 0.6 for permuted GE layers of the set with maximal GE in the ectoderm layer, *p* = 0.0012.

### Genome architecture and transcriptome architecture

The genome architecture is characterised by the distance between each gene *i* and its nearest neighbour, *d*_*i*_ = min(|TSS_*i*−1_ − TSS_*i*_|, |TSS_*i*+1_ − TSS_*i*_|), where TSS is the most 5′ annotated transcription start site and genes have been ordered by their TSS. The transcriptome architecture is similarly defined, but we exclude genes whose expression level is below a threshold when calculating distances. Threshold values were from [−3, −2, −1, 0, 1, 2, 3] in units of log2 RPKM.

To evaluate the differences in nearest neighbour distances for DEG and SEG gene sets we used the Mann-Whitney test at significance *p* < 0.05.

### GO analysis

We used four different GO enrichment tools: panther [108], Gorilla [109], goliath, and g:Profiler [110] to evaluate the categories enriched amongst similarly and differentially expressed genes. Default parameters were applied. All four methods were consistent and the results from g:Profiler are reported in Table 1, Additional file 1: Table S2 and Fig. S2E.

### 3D organisation: TADs and genes

TAD annotations were used from [29, 33]. We computed the numbers of SEGs and DEGs within each TAD. We then computed the lengths of DEG- and SEG-containing TADs, and gene density within them as the number of genes divided by the length of the TAD.

We compared gene density and lengths of DEG and SEG-containing TADs and tested significance of their differences with the Mann-Whitney test at *p* < 0.05 level.

### Chromatin accessibility

To identify accessible regions, we divided the genome into non-overlapping 100-bp windows and we computed a number of GC dinucleotides in each window. We also computed the accessibility level within each window, *Aj* (*j* = 1,2,3 for the three lineages) as a percentage of all GC-methylated dinucleotides counts divided by the total number of GC dinucleotides.

Similarly to DEGs vs SEGs, we applied the Dominance index [101] strategy to call differentially accessible regions (DARs) for ectoderm, endoderm, and mesoderm and similarly accessible regions (SARs) within each 100-bp window:

$$Da={\text{max}}(A_i)/\sum A_i,$$

where *A1*, *A2*, and *A3* are the accessibility levels for the three lineages, defined as the fraction of accessible (methylated here) GC to all GC in the window [47].

We selected windows with high dominance of one lineage level, A*, over other lineage levels. The dominance threshold *D*_*high*_ = *0.6* was selected according to [102–104]. We required *D*_*low*_ = *0.34* to define SARs. We computed the permutation test at *p* < 0.0001 level to ensure significance of the dominance threshold. All further analysis (see sections about linking DEGs and DARs) was done for data collected by Dominance index strategy.

#### QC of accessibility data and the NMT_seq GC bias

At the first step, we filtered out the windows with insufficient coverage (fewer than 25 reads), to avoid calling low-confidence DARs. We computed the number of GCs in non-overlapping 100-bp windows throughout the genome, based on sequenced read data. Note that for NOME_seq technology, methylated GC means that the area around it is occupied by a nucleosome. We denote the number of reads in each window by *A*_*i*_ and the number of GCs by *C*_*i*_. We filtered out the relative coverage-unbalanced and GC-number-unbalanced windows. The set of QC tests/filters and parameters are as follows:

- *A*_*i*_ > 25 and *A*_*i*_/*C*_*i*_ > 2
- Relative coverage balance between lineages, adjusted for the cell number in each set. We required that variability between the three values for coverage in a window is less than one standard deviation over mean:
  *d*_*ij*_ = *(cov*_*i*_* − cov*_*j*_*), i,j in {1,2,3}* are differences between coverages for three lineages in a window
    $$|max |{d}_{ij}| - mean({cov}_{1},{cov}_{2},{cov}_{3})| < std({cov}_{1},{cov}_{2},{cov}_{3})$$
- GC number balance between lineages within corresponding windows. We required that variability between the three values for GC numbers in a window is less than one standard deviation over mean.
  *dif_numGC*_*ij*_ = *(numGC*_*i*_* − numGC*_*j*_*), i,j in {1,2,3}* are differences between GC numbers for three lineages in a window
    $$|max |dif\_numG{C}_{ij}| - mean(nG{C}_{1},numG{C}_{2},numG{C}_{3}) | < 3*std(numG{C}_{1},numG{C}_{2},numG{C}_{3})$$

For all windows, both DARs and SARs, we computed the medians of GC counts (Additional file 1: Fig. S5B). The SAR set was subdivided into highly accessible (HA) and low accessible (LA), HA accessibility threshold is more than 35% of accessibility in a window, while LA is less or equal 35%. The distributions of retained and filtered out windows depending on GC counts and minimal coverage threshold are shown in Additional file 1: Fig. S5A.

#### Count of DAR and SARs around TSS (normalised by number of expressed genes) and H3K27ac

We analysed the spatial distribution of accessible chromatin regions (ACR) around TSSs for both DEGs and SEGs. We fixed the vicinity of a gene to be XK bp (X is 20,40,80,120,…300,400, ‘K’ means kilobases here), then ACR regions were counted within (−XK,XK) intervals around TSS. The value was then normalised by the number of genes in the region of interest.

We introduced an accessibility index to measure and visualise distribution of DARs and SARs around DEGs and SEGs:

$$\mathrm{Accessibility \,Index}=\frac{{N}_{AR}\left({V}_{ij}\right)/{N}_{g}\left({V}_{i}\right)}{{N}_{EG}},$$

where ${N}_{AR}({V}_{ij})$ = *number of accessible regions in bin j of vicinity *${V}_{i}$*,*${V}_{i}$ = *vicinity of (differentially or similarly) expressed gene i, centred at the transcription start site of that gene;*${N}_{g}({V}_{i})$ = *number of expressed genes in vicinity *${V}_{i}$*;*${N}_{EG}$= *number of (differentially or similarly) expressed genes in the given set.*

Since we defined DARs in a lineage-specific way, the mesoderm-accessible DARs were counted around mesoderm upregulated DE genes, and correspondingly, ectoderm and endoderm-accessible DARs were counted around ectoderm or endoderm upregulated DE genes. We computed DAR/SAR frequency in a large fixed vicinity of DEGs/SEGs TSS, the size of vicinities mimicking the TAD’s size ranges, from 20 K, 40 K,….up to 1 MB for some chromosomes.

We performed a permutation test to assess lineage specificity by swapping DAR sets and comparing the resulting clustering distribution around non-matched DEGs TSS. We computed a background frequency of DARs for all genes within the genome.

We used a set of Chip-Seq-derived lineage-specific H3K27ac (annotation and data from [111]), for the same E7.5 day of embryo development. We computed a frequency of matched DARs around the H3K27ac. To test the specificity of DAR clustering around lineage-specific H3K27ac, we ran a permutation test swapping DARs across lineages. We applied the widely used differentially methylated regions finding method Defiant [112] and compared with our approach by using H3K27ac sets as markers for optimal performance. Our method was more sensitive: we retrieved twice as many H3K27ac peaks (for the roughly same amount of DhMR regions), compared to Defiant.

### Correlation of chromatin accessibility with gene expression

#### Chromatin abundance coefficient

To correlate DAR/SAR frequency to GE level, we developed the chromatin abundance coefficient (CAC) for a set of genes. To compute CAC, the number of open chromatin windows (DARs or SARs) was calculated across the TSS vicinity *V*_*i*_ for each gene *g*_*i*_ in a set first, *N(V*_*i*_*)*. Then the number is normalised by the count of expressed genes in this vicinity, *Ng(V*_*i*_*)* (normalised accessible region frequency):

$$\mathrm{normalised\, AR\, frequency\, in\, }Vi = N({V}_{i}) / Ng({V}_{i})$$

It is then compared with the average gene expression (within the same normalised frequency counts) across genes having *GE*_*i*_, by computing a Pearson correlation coefficient:

$$\mathrm{CAC }= Pearson\, correlation \,(N({V}_{i}) / Ng({V}_{i}), \,mean(G{E}_{i}))$$

If the CAC value is positive and high (*R*^2^ > 0.7), we define the corresponding sets of genes and DARs as linked.

#### Determining ‘domains of influence’ for DEGs-DARs, and linked gene-DAR combinations

We searched what TSS vicinity ranges give high or low correlations with GE, and at what vicinity the correlation vanishes. Assuming that the majority of DARs within the vicinity are associated with their corresponding genes, we identify a vicinity of TSS giving maximal correlation. The zone of maximal influence, Z*, is defined as below:

$$Z^* = argmax (Rk={\text{CAC}}({\text{Zk}}) | Zk \subset \{Z1,Z2,...Zi\})$$

where *Rk* and *Zk* = {TSS vicinities of a gene set, having DAR within them}, *Rk* = correlation coefficient (for number of DARs from *Zk* and GE of genes from a given gene set). We compute the ‘Gene Expression-DARs frequency’ correlations for different upstream/downstream fixed zones. We computed the CAC correlation for SEGs and SARs in a similar way.

#### H3K27ac and DEGs

We computed the normalised frequency of H3K27ac enhancers (annotation from [18], data from [36]) around the TSS vicinity of DEGs, similarly to DEG-DARs above. We compared these frequencies with GE expressions to determine if there is a positive correlation. We computed ‘zones of enhancer influence’ for the H3K27ac sets in a similar way to above.

#### Algorithm to link DEGs and DARs

We searched a range of vicinities for the one giving maximal CAC (while keeping high enough DAR density) correlation of average gene expression and DAR’s frequency. We do it separately for each corresponding lineage and chromosome. We assume that the majority of these maximally correlated DARs are putative enhancers for their target genes within a given region of high correlation (domain of influence).

The algorithm to link DEGs and DARs is as follows:

1. Retrieve the TSS vicinity zone giving maximal CAC correlation coefficient.
2. Retrieve the corresponding genes, TADs (containing these genes) and their linked DARs within this zone, excluding pairs that are found in different TADs.

We retrieved linked DARs for each gene from DEG to make a catalogue (Additional files 2, 3 and 4) of differentially expressed genes and their differentially accessible chromatin regions, sitting in the corresponding TADs. The requirement of exclusion of DARs that are found in different TADs made the majority of DARs localised in the 100 K vicinity of TSS, with only around 15% of them spreading further than 100 kB.

#### DhMR and ShMR detection, and clustering around DARs, H3K27ac and SARs

We applied the same procedures to find DhMRs as we did for DARs, but with a wider window of 500 bp. Likewise to DARs and SARs, we applied the Dominance index [101] strategy to call differentially hypomethylated regions (DhMRs) and similarly DNA hypomethylated regions (ShMRs) within each 500-bp window. Dominance index of methylation level across the three per-lineage values, within each window:

$$Dom =\mathrm{ max}({M}_{i})/\sum {M}_{i}.$$

where *M*_*1*_*,M*_*2*_*,M*_*3*_ are the fraction of methylated CpG within a window in ectoderm, endoderm and mesoderm tissues. We used a threshold *Dom* = *0.6* [102, 103] to classify a DhMR as differentially hypomethylated, and *Dom* = *0.34* to classify ShMR as similarly hypomethylated, likewise identification of DAR/SAR. We fixed the vicinity = 10,000 bp around DAR and SAR central point, and computed the number of DhMRs and ShMRs within this range. We computed the Jaccard index for DARs and DhMR and for ShMRs and SARs. We ran a set of permutation tests to ensure significance of DhMR/ShMR clustering at *p* < 0.01.

### Difference in DEG and SEG regulation with respect to TF distal and proximal binding

#### Design: choosing the regions for TFBS enrichment analysis

To search for TF binding motifs, we pooled all DARs/DhMRs sequences (putative enhancers) into three lineage-specific groups (Additional file 1: Fig. S7A top: pink, green, blue). We also took DNA sequences from 100 bp upstream and 50 bp downstream of TSS [113] to form three pools of core promoters (DEG CPs). Similarly, SARs were combined into one group and promoters of SEGs into another (SEG CPs) (Additional file 1: Fig. S7A bottom: orange). We investigated the motif enrichment of DNA within these eight groups (Additional file 1: Fig. S7B), and enriched motifs were ranked based on the *p*-values of their overrepresentation. Next, we filtered corresponding TFs by their expression in our transcriptome data sets.

We hypothesised that promoter-enhancer specificity is manifested by higher degree of similarity within than between regulatory neighbourhoods, e.g. DEG enhancers are more similar to DEG promoters than to SEG promoters. To test how different is DEG’s and SEG’s promoter-enhancer specificity with respect to TF binding, we computed RMSE-based weighted similarity scores within and between the DEG and SEG motif repertoires. We also computed promoter-promoter and enhancer-enhancer specificity (similarity scores, defined as rmse = root mean square error) within and between the DEG and SEG neighbourhoods separately (Additional file 1: Table S3).

We test the hypothesis that TF binding is similar between DEGs and SEGs. Our alternative hypothesis is that it is different (therefore specific, see definition [3]).

We define a region to be a ‘putative DEG enhancer’ if it is lineage-specific, accessible and hypomethylated. There are 960 ectoderm-specific, 5230 endoderm-specific and 1382 mesoderm-specific putative enhancers. We define a region to be ‘putative SEG enhancers’ if it is similarly accessible and hypomethylated across all three lineages (6354 regions). Collectively, we have four sets of putative enhancers: three sets of DEGs, and one set of SEG’s enhancers (Additional file 1: Fig. S7B). In line with [17], we define ‘core promoter (CP)’ as the region [−100, 50] relative to the TSS. We have four DEG CP sets and one SEG CP set (Additional file 1: Fig. S7B). We measure the similarity of two sets of regions based on the similarity of their TFBS repertoires.

We say that a local set, consisting of a promoter with associated putative enhancers (or other promoters), which are likely to be 3D-close, constitutes a *regulatory neighbourhood* of the given promoter.

### Obtaining enriched motifs (TFs repertoires) within putative enhancers and promoters

We ran five motif enrichment tools (each with default parameters) to check a consistency of motif search. We use the intersection of motif searches: Homer [56], RSAT [114], GREAT [115], DMINDA2 [116], AME meme suit [117]—on our eight sets of putative enhancer and core promoters (the list of these motifs is presented in Additional file 5), and obtained eight lists of significantly enriched TF motifs, Additional file 1: Fig. S7A, with a *p*-value < 0.001. Each motif within a list is ranked in ascending order based on *p*-values (Additional file 1: Fig. S7C).

We filtered the lists of enriched motifs by their gene expression in our data (GE in log2(RPKM), GE > 0), discarding around 25% of motifs corresponding to non-expressed genes. We sorted filtered TF motifs according to where their corresponding genes were expressed: predominantly in one lineage (DEG) or almost evenly across all three (SEG).

### Measurement of difference of TF repertoire

We measure TF difference by overall weighted motif similarity between each pair of the eight TF lists. We extracted the union of all motifs within all eight region sets, which is our main feature. It includes 277 TFBS motifs (Additional file 5). For each set (e.g. ectoderm enhancers), we ranked motifs according to their frequency. Because the lists of motifs are of different length, we ranked motifs by quartiles of the list, e.g. 1,2,3,4. For example, Maz and Zn281 are ranked as 1 (in first list frequency quartile) in ectoderm enhancers, but as 2 in ectoderm promoters, while Po3f1 is ranked 2 in ectoderm enhancers, and ranked 0 (not present) in promoters. We represent each set of enhancers and CPs as a feature-vector of its list’s rank-values for each feature entry or zeros, if a motif is missing. We compute a pairwise weighted similarity between ranked lists of TF motifs (feature-score vectors) as RMSE of fitting feature-scores to the line $y=x$ (a model assuming the lists were equal in frequency) for each two vectors:

$$RMSE =\sqrt{{\sum }_{i=1}^{n}{({\widehat{yi}}-fi)}(\widehat{yi}-fi){/n}}$$

where $\widehat{{y}_{i}}$ are the points of the line $y=x$, and $fi$ are the motif’s feature-scores for both vectors. In this way, we account for motifs that are absent in one of the lists, and on the degree of score (reflecting motif’s frequency) for those which are common between the lists. The higher the RMSE, the smaller the similarity between two TF lists. We first computed overall similarity within and between DEG and SEG enhancers and promoters as a median value across pairwise similarity for each lineage pair (Table S3). Then we computed the difference between overall similarities of DEG-DEG and DEG-SEG sets. We tested how significant this difference is by both *t*-test and permutation tests. We ran a set of rank permutation tests within each list of motifs per promoter or enhancer regions to check a significance of similarity difference. We permuted each motif’s ranks and each motif’s occurrence, simulating DNA sequence shuffling while preserving the same GC-richness (by drawing from GC-rich motifs for SEG enhancers and promoters).

We computed overall similarity between and within enhancers and CPs: we compared core promoters of DEGs and SEGs, then corresponding putative enhancers, and finally, promoters-enhancers (Table S3).

### Retrieving motifs contributing to enhancer-promoter difference between DEGs (developmental) and SEG (housekeeping) regulation

We defined two distinct sets of motifs, common between enhancer-core promoters within DEGs and within SEGs, by selecting motifs that occurred across lists of DEG and SEG regulatory regions at high (the first *p*-value quartile) scores. We compared them with those found in [51].

### In-depth analysis of motifs’ features contributing to sequence-encoded enhancer-promoter specificity

This analysis includes the following features of motifs: nucleotide content, complexity, motif length.

We define a single-nucleotide content, (Pa,Pc,Pg,Pt), of a motif as a proportion of occurrence A,C,G or T in motif’s sequence, such as

$$Px= count( x)/length(motif), x\mathrm{\, is\, from \,}\{{\text{A}},{\text{C}},{\text{G}},{\text{T}}\}$$

A di-nucleotide content is a count of each adjacent pair divided by the length of the motif −1.

$$Pxy= count( xy)/(length(motif)-1), \,xy\mathrm{\, is \,from\, }\{{\text{AA}},{\text{AC}},{\text{AG}},{\text{AT}}\dots .{\text{TA}},{\text{TC}},{\text{TG}},{\text{TT}}\}$$

A di-nucleotide entropy of a motif is given by the formula below

$$entropy=-\sum Pxy(log2(Pxy))$$

where summation goes over all nucleotide adjacent pairs in a motif. We take the entropy value as a measure of a motif’s complexity.

We studied the motifs specific to SAR-SEG promoters and enhancers (intersection of high-ranked motifs) and specific for DAR-DEG promoters and enhancers (union of all three DAR-DER high-ranked motif intersections). We ran non-parametric statistical tests (Wilcoxon and Kruskal–Wallis) to infer significance of the differences.

We also computed the percentage of known TFs [75] in DEGs and SEGs, and average percentage across all mouse genes. We used a one tailed Fisher test to determine significance.

### Difference within DEG’s putative enhancers: inferring lineage-specific driver TFs

We searched for TF motifs which could contribute to differences in developmental regulation across lineages. We focus on DEG’s putative enhancers, as in Additional file 1: Fig. S7B bottom. We defined lists of distinct motifs within DEG enhancers. We retrieved lineage-specific-enriched TF motif repertoires, and the most significant ones are listed in Fig. 5B.

### Confirming per-lineage difference and function by literature search

We compared our results of enriched TFs in Fig. 5B with TFs which are reported to be important in lineage specification and pattern formation. We checked if the TFs—producing genes for the enriched motifs—were expressed mostly in DEGs or in SEGs. We confirmed from the literature what motifs were pioneer (happened to be DEG-produced), and which were not (SEG-produced) (Additional file 1: Table S4 for references).

### Data used

Pseudo-bulk per ectoderm, endoderm, mesoderm for transcriptome, chromatin accessibility, and Methylome for E7.5, from scNMT_seq, as in [18]. The set of H3K27ac is from [36] and H3K4me3 is from [18]. Raw sequencing data together with processed files (RNA counts, CpG methylation reports, GpC accessibility reports) are available in the Gene Expression Omnibus under accession number GSE121708. Data can be downloaded from ftp://ftp.ebi.ac.uk/pub/databases/scnmt_gastrulation.

### Supplementary Information

**Additional file 1: Fig. S1.** Differences in gene expressions within DEGs and between DEG and SEG. (A) Endoderm-expressed DEGs are significantly higher expressed than ectoderm and mesoderm-specific DEGs (Kruskal-Wallis *p* < 2.2e-16), while ectoderm and mesoderm-specific DEGs do not differ significantly by gene expression (Kruskal-Wallis *p* = 0.38). (B) SEGs are significantly higher expressed than DEGs (Wilcoxon, *p* = 2.2e-16). **Fig. S2.** SEG and DEG features: dynamics of GE, CG content and relation to HKG. (A) Intersection of SEGs and HKG: 61% of SEGs (right column) are known as HKG. (B) SEG gene expression from day E4.5 to E7.5: not changing, expressed all the time. (C) GE for DEGs due to their respective trajectory: ectoderm DEGs are already expressed early days, and gradually increase expression up to day E7.5; endoderm and mesoderm genes DEG genes are lower expressed in days E4,5 E5.5, significantly decline day E6.5 and highly expressed day E7.5. (D) Percentage of CG-richness in DEGs and SEGs promoters (*p* < 0.01, Kruskal Wallis test). (E) Counts of protein-containing complex genes and anatomical entity genes in DEGs and SEGs, GO cellular components. A one sample t-test on the proportion of protein-containing complex to anatomical entity (red/violet) shows significant difference in the proportion between DEGs and SEGs, *p* = 0.0039. **Fig. S3.** DEGs and SEGs subsets with same GE range still have main features separating them: (A) All genes. Illustration of difference in GE between ectoderm (green) and mesoderm (pink) DEGs, compared to SEG GE (orange): higher GE for SEGs than DEGs; (B)Subsets of genes: we take only those genes which have the same GE range for SEGs and DEGs; illustration of corresponding lineage GE similarities for (expressed) genes. © CG content of all DEG SEG sets. (D) CG content of DEG SEG subsets from B. (E) Distances to the nearest expressed gene for DEGs and SEGs per GE thresholds (anova, *p* = 5.57e-15, threshold GE). (F) DEGs are further away from other expressed genes than SEGs (anova, *p* = 1.52e-84, gene set). (G) Line plots for mean GE (Y-axis) depending on thresholds (X-axis) per each lineage (colored): all lineages are statistically different from each other with respect to the distance to the nearest gene. **Fig. S4.** Illustration of four TADs containing DEGs and SEGs. (A) SEGs Prdx1 and Rps8 (left and right borders) + SARs and many SEG-type genes between them. (B) SEGs Ccdc142 (centred) + SARs and many SEG-type genes between them. (C) Shh loci with known enhancers (green) and DARs (black). (D) Cxcl12 ectoderm expressed gene, isolated within its TAD (other genes are not expressed) with marked H3K27ac and DARs. **Fig. S5.** DAR’s QC: Filtering by coverage removes GC-bias of accessibility data. (A) Red is GC distribution of filtered out DAR’s regions. Blue is the GC-distribution of remaining accessibility windows. The remaining window distribution is a fair approximation of genome-wide GC-distribution. (B) Box plots showing median values and outliers for DARs, SARs (low SARLA, and high SARHA) and genome-wide (ALL). (C) DAR occupancy of TSS vicinity permutation test. Permutation test showing that peaks and valleys of DARs around DEG TSS are not by chance, where chance is represented by random and uniform distribution of the same number of regions around TSS within the same vicinity. Here only 1 or2 histogram values out of 1000 simulated histograms reach any of non-central peaks, therefore *p* < 0.005. **Fig. S6.** Enhancer-promoter specific sequence features (DAR-DEG dark cyan, SAR-SEG orange) which are significantly different between DEGs and SEGs: complexity, A-nucleotide content, length. **Fig. S7.** Data design illustration: (A) Regulatory neighbourhoods: sets of DEGs SEGs with their putative enhancers (DARs/DMRs/SARs/SMRs) shown as ovals, and core promoters, as circles: (top) developmental neighbourhood; (bottom) housekeeping neighbourhood. (B) Mapping DNA sequences of DARs/DMRs and core-promoters into lists of enriched TFBS motifs within them (coloured rectangles); (C) Illustration of a list with enriched TF motifs, represented as rectangle. **Fig. S8.** Schematic representation of developmental enhancer- promoter activation with (a) binding DEG-produced TF to nucleosome (b) recruiting other TFs and transcription machinery (including SEG-produced TFs); (c) bridging corresponding target gene’s promoter by SEG-produced TFs, which all leads to the gene transcription. **Table S1.** Known developmental marker genes [120–122]. **Table S2.** GO MF Terms for DEGs and SEGs. **Tables S3 extended.** Specificity of enhancers-promoters between DEGs and SEGs regulation. Table S3.1 extended: pairwise rmses between promoters. Table S3.2 extended: pairwise rmses between enhancers. Table S3.3 extended: pairwise rmses between enhancers and promoters. **Table S4.** Pioneer and non-pioneer TF role, mentioned in a literature.

**Additional file 2.** Bed files of DARs in Ectoderm, filtered by corresponding TADs borders.

**Additional file 3.** Bed files of DARs in Endoderm, filtered by corresponding TADs borders.

**Additional file 4.** Bed files of DARs in Mesoderm, filtered by corresponding TADs borders.

**Additional file 5.** TFBS motif names, common for all searched databases.

## Data Availability

The dataset supporting the conclusions of this article is included within the article and its Additional files 2, 3 and 4 (bed files of DARs catalogue linked to their target genes). The NOME-seq data set, used in the manuscript, is available in the Gene Expression Omnibus under accession GSE121708 [118]. Processed data can be downloaded from ftp://ftp.ebi.ac.uk/pub/databases/scnmt_gastrulation [18]. The codes supporting the conclusions of this manuscript are available at https://github.com/irinaabnizova/joint-multiomic-analysis [119].

## Abbreviations

TF: Transcription factor
TFBS: Transcription factor binding sites
DNA: Deoxyribonucleic acid
TSS: Transcription start site
DEG: Differentially expressed gene
SEG: Similarly expressed gene
TAD: Topologically associated domain
HKG: Housekeeping genes
GO: Gene ontology
CG content: Percentage of nucleotides A,C,G or T
CG-rich: High CG count
A-rich: High A nucleotide count
RNAPII: RNA polymerase II
scNMT-seq: Single-cell nucleosome, methylation and transcription sequencing
DAR: Differentially accessible region
SAR: Similarly accessible region
H3K27ac marks: An epigenetic modification to the DNA packaging protein histone H3, it indicates acetylation of the lysine residue at N-terminal position 27 of the histone H3 protein
H3K4me3 histone marks: An epigenetic modification to the DNA packaging protein Histone H3 that indicates tri-methylation at the 4th lysine residue of the histone H3 protein
ChIP-seq: Chromatin immunoprecipitation (ChIP) assays with sequencing
CAC: Chromatin abundance coefficient
DhMR: Differentially hypomethylated methylated region (a small ‘h’ in DhMR denotes low methylation level)
ShMR: Similarly hypomethylated region (ShMRs)
MAD-score: Median absolute deviation is a robust measure of the variability of a univariate sample of quantitative data
Dom: Dominance index
H0: Null hypothesis
H1: Alternative hypothesis
RPKM: Reads per kilobase per million
ACT: Accessible chromatin regions
GE: Gene expression
CP: Core promoter
RMSE: Root mean square error
