# Supplementary material for: Integrative analysis of transcriptomic and epigenomic data reveals distinct patterns for developmental and housekeeping gene regulation
Source: BMC Biol. 2024 Apr 10;22:78. doi: 10.1186/s12915-024-01869-2 (PMC11005181; doi:10.1186/s12915-024-01869-2)
Supplement: Supplementary file 1 — Additional file 1: Fig. S1. Differences in gene expressions within DEGs and between DEG and SEG. (A) Endoderm-expressed DEGs are significantly higher expressed than ectoderm and mesoderm-specific DEGs (Kruskal-Wallis p < 2.2e-16), while ectoderm and mesoderm-specific DEGs do not differ significantly by gene expression (Kruskal-Wallis p = 0.38). (B) SEGs are significantly higher expressed than DEGs (Wilcoxon, p = 2.2e-16). Fig. S2. SEG and DEG features: dynamics of GE, CG content and relation to HKG. (A) Intersection of SEGs and HKG: 61% of SEGs (right column) are known as HKG. (B) SEG gene expression from day E4.5 to E7.5: not changing, expressed all the time. (C) GE for DEGs due to their respective trajectory: ectoderm DEGs are already expressed early days, and gradually increase expression up to day E7.5; endoderm and mesoderm genes DEG genes are lower expressed in days E4,5 E5.5, significantly decline day E6.5 and highly expressed day E7.5. (D) Percentage of CG-richness in DEGs and SEGs promoters (p < 0.01, Kruskal Wallis test). (E) Counts of protein-containing complex genes and anatomical entity genes in DEGs and SEGs, GO cellular components. A one sample t-test on the proportion of protein-containing complex to anatomical entity (red/violet) shows significant difference in the proportion between DEGs and SEGs, p = 0.0039. Fig. S3. DEGs and SEGs subsets with same GE range still have main features separating them: (A) All genes. Illustration of difference in GE between ectoderm (green) and mesoderm (pink) DEGs, compared to SEG GE (orange): higher GE for SEGs than DEGs; (B)Subsets of genes: we take only those genes which have the same GE range for SEGs and DEGs; illustration of corresponding lineage GE similarities for (expressed) genes. © CG content of all DEG SEG sets. (D) CG content of DEG SEG subsets from B. (E) Distances to the nearest expressed gene for DEGs and SEGs per GE thresholds (anova, p = 5.57e-15, threshold GE). (F) DEGs are further away from other [file 12915_2024_1869_MOESM1_ESM.docx]

Additional file1

## Supplementary section

#### Supplementary figures


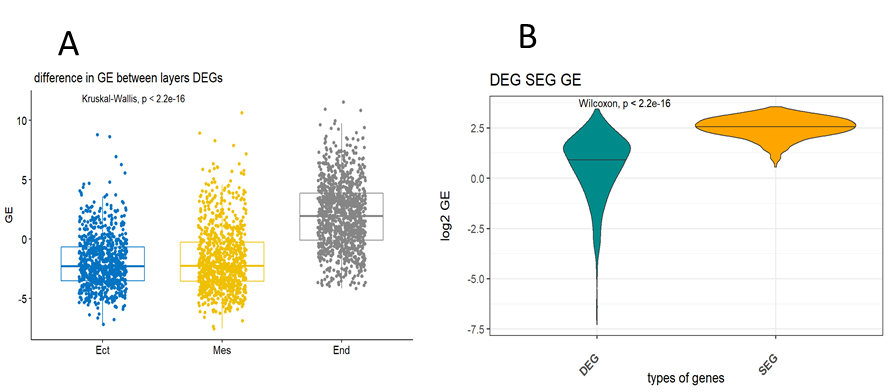


**Fig. S1** Differences in gene expressions within DEGs and between DEG and SEG

1. Endoderm-expressed DEGs are significantly higher expressed than ectoderm and mesoderm-specific DEGs ( Kruskal-Wallis p<2.2e-16), while ectoderm and mesoderm-specific DEGs do not differ significantly by gene expression (Kruskal-Wallis p=0.38)
2. SEGs are significantly higher expressed than DEGs (Wilcoxon, p=2.2e-16)


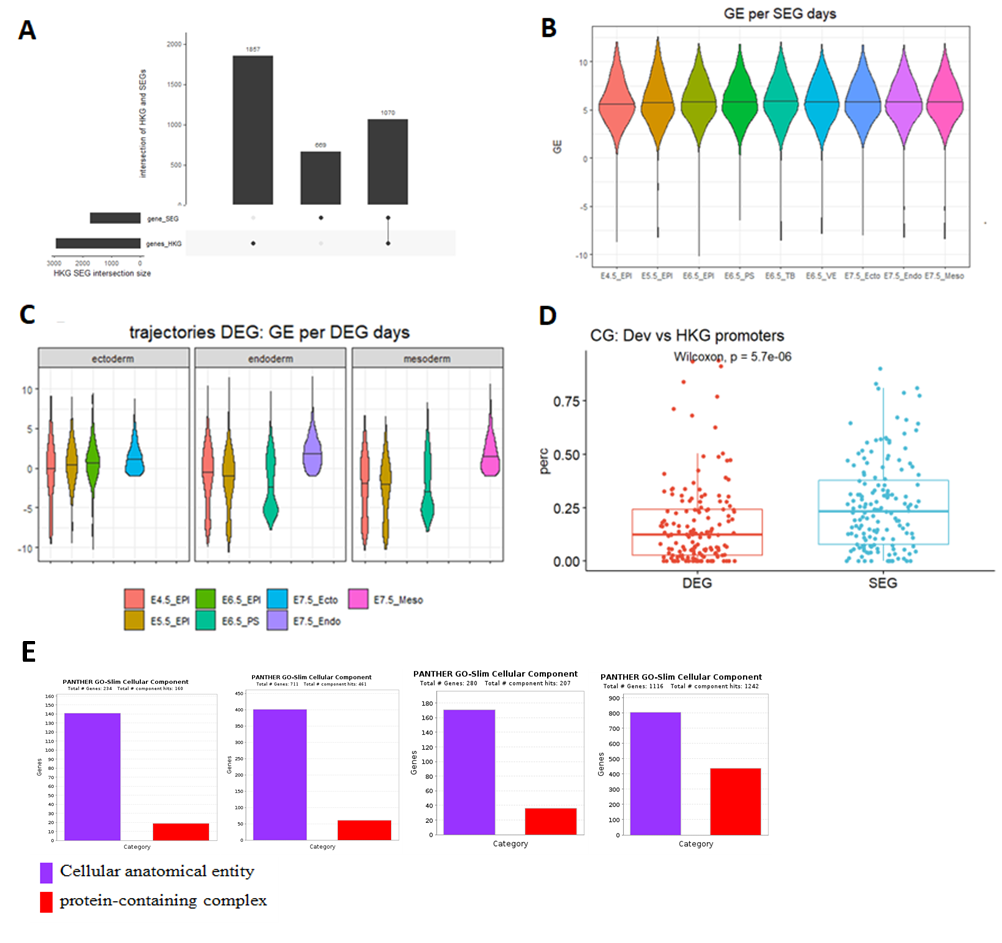


**Fig. S2.** SEG and DEG features: dynamics of GE, CG content and relation to HKG

(A) Intersection of SEGs and HKG: 61% of SEGs (right column) are known as HKG.

(B) SEG gene expression from day E4.5 to E7.5: not changing, expressed all the time.

(C) GE for DEGs due to their respective trajectory: ectoderm DEGs are already expressed early days, and gradually increase expression up to day E7.5; endoderm and mesoderm genes DEG genes are lower expressed in days E4,5 E5.5, significantly decline day E6.5 and highly expressed day E7.5.

(D) Percentage of CG-richness in DEGs and SEGs promoters (p<0.01, Kruskal Wallis test).

(E) Counts of protein-containing complex genes and anatomical entity genes in DEGs and SEGs, GO cellular components. A one sample t-test on the proportion of protein-containing complex to anatomical entity (red/violet) shows significant difference in the proportion between DEGs and SEGs, p=0.0039.

**
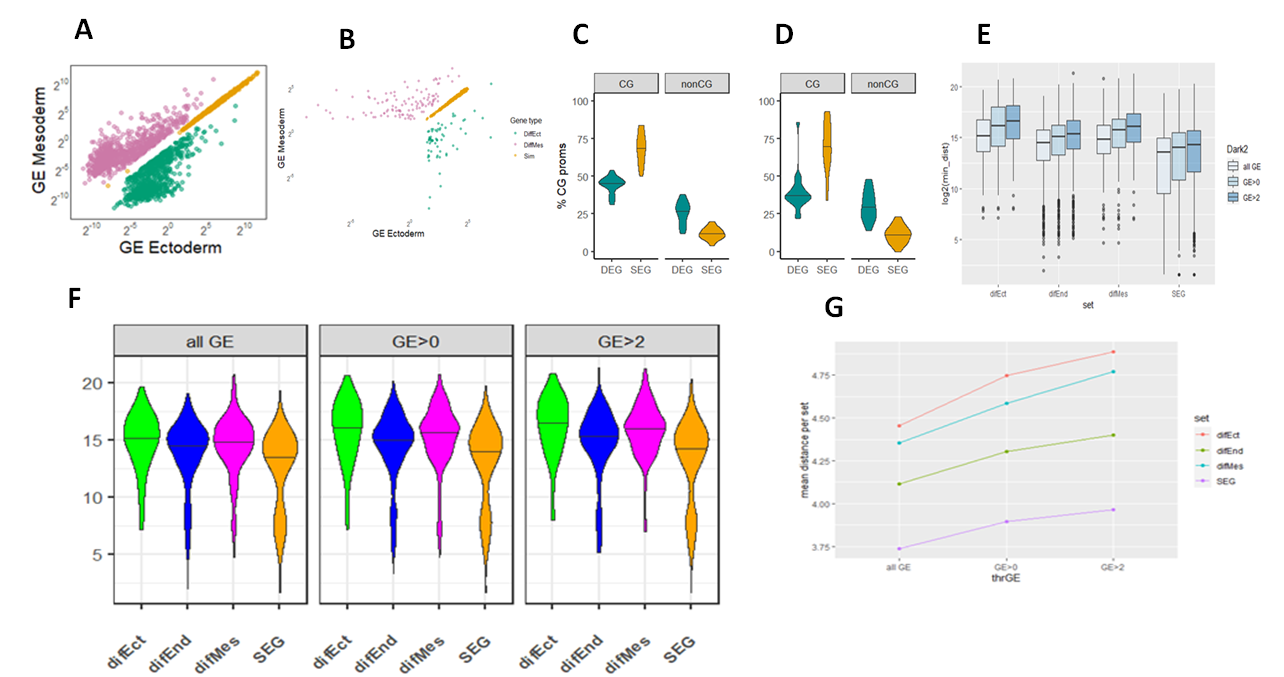
**

**Fig. S3.** DEGs and SEGs subsets with same GE range still have main features separating them:

(A)All genes. Illustration of difference in GE between ectoderm(green) and mesoderm(pink) DEGs, compared to SEG GE (orange): higher GE for SEGs than DEGs;

(B)Subsets of genes: we take only those genes which have the same GE range for SEGs and DEGs; illustration of corresponding lineage GE similarities for (expressed) genes

© CG content of all DEG SEG sets

(D) CG content of DEG SEG subsets from B.

(E) Distances to the nearest expressed gene for DEGs and SEGs per GE thresholds (anova, p= 5.57e-15, threshold GE)

(F) DEGs are further away from other expressed genes than SEGs (anova, p= 1.52e-84, gene set)

(G) Line plots for mean GE (Y-axis) depending on thresholds (X-axis) per each lineage (colored): all lineages are statistically different from each other with respect to the distance to the nearest gene

ANOVA Table (type II tests) results for Fig S2 E,F,G

Effect DFn DFd F p p<.05 ges

1 set 3 5140 135.593 1.52e-84 * 0.073000

2 thrGE 2 5140 33.031 5.57e-15 * 0.013000

3 set:thrGE 6 5140 0.665 6.78e-01 0.000776


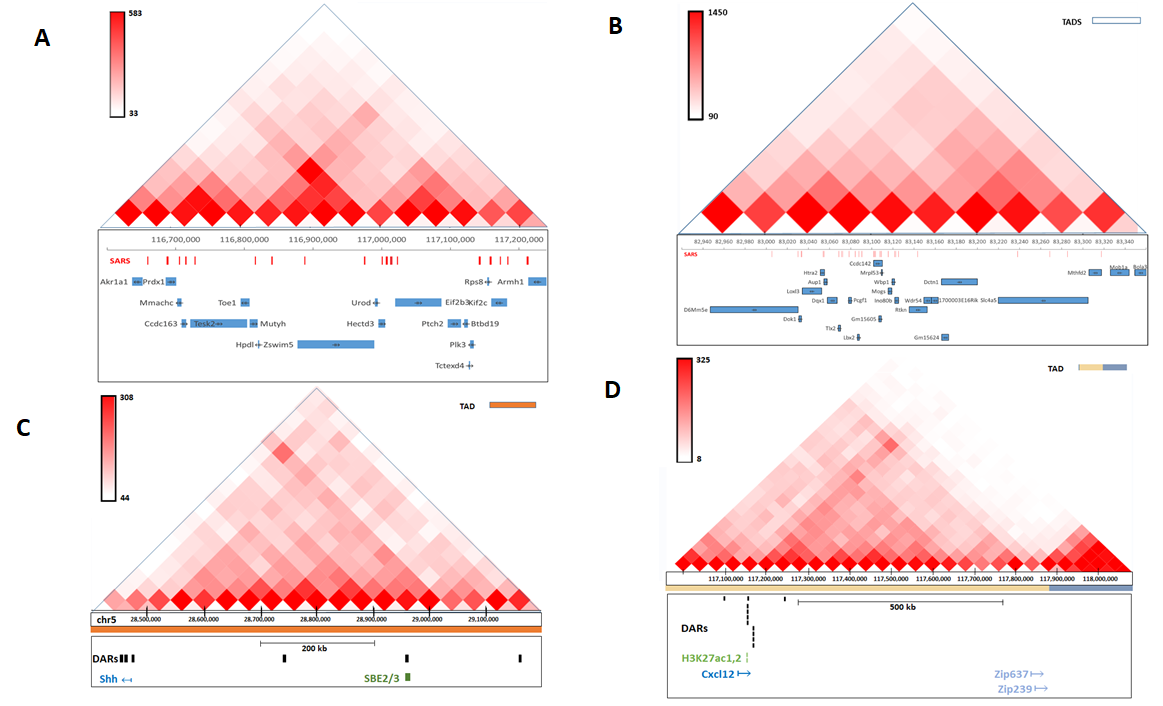


**Fig. S4.** Illustration of four TADs containing DEGs and SEGs

1. SEGs Prdx1 and Rps8 (left and right borders) + SARs and many SEG-type genes between them
2. SEGs Ccdc142 (centred) + SARs and many SEG-type genes between them
3. Shh loci with known enhancers (green) and DARs (black).

(D) Cxcl12 ectoderm expressed gene, isolated within its TAD (other genes are not

expressed) with marked H3K27ac and DARs.


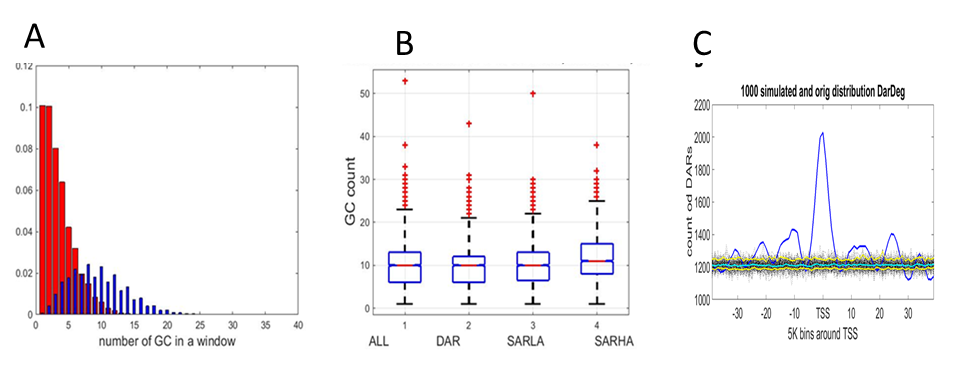


**Fig. S5.** DAR’s QC: Filtering by coverage removes GC-bias of accessibility data

1. Red is GC distribution of filtered out DAR’s regions. Blue is the GC-distribution of remaining accessibility windows. The remaining window distribution is a fair approximation of genome-wide GC-distribution.
2. Box plots showing median values and outliers for DARs, SARs (low SARLA, and high SARHA) and genome-wide (ALL).
3. DAR occupancy of TSS vicinity permutation test. Permutation test showing that peaks and valleys of DARs around DEG TSS are not by chance, where chance is represented by random and uniform distribution of the same number of regions around TSS within the same vicinity. Here only 1 or2 histogram values out of 1000 simulated histograms reach any of non-central peaks, therefore p < 0.005.


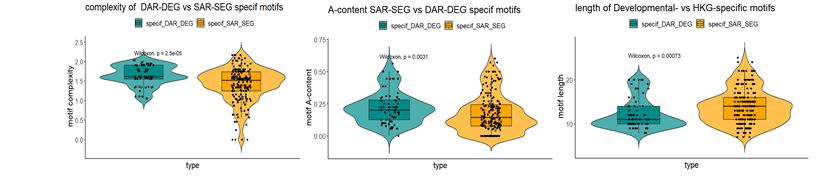
**Fig S6.** Enhancer-promoter specific sequence features (DAR-DEG dark cyan, SAR-SEG orange) which are significantly different between DEGs and SEGs : complexity, A-nucleotide content, length


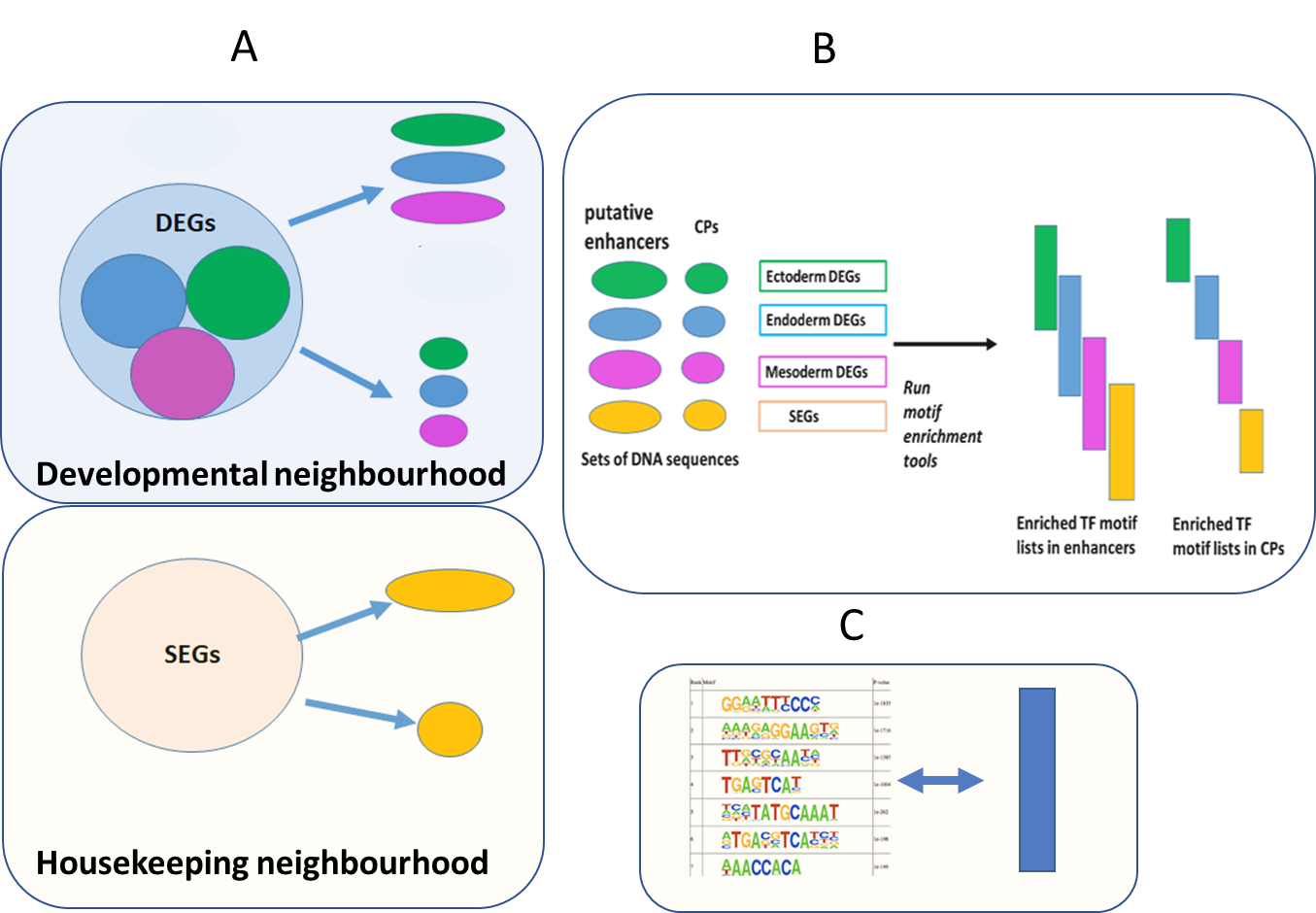


**Fig. S7.** Data design illustration:

1. Regulatory neighbourhoods: sets of DEGs SEGs with their putative enhancers (DARs/DMRs/SARs/SMRs) shown as ovals, and core promoters, as circles: (top) developmental neighbourhood; (bottom) housekeeping neighbourhood.
2. Mapping DNA sequences of DARs/DMRs and core-promoters into lists of enriched TFBS motifs within them(coloured rectangles);
3. Illustration of a list with enriched TF motifs, represented as rectangle


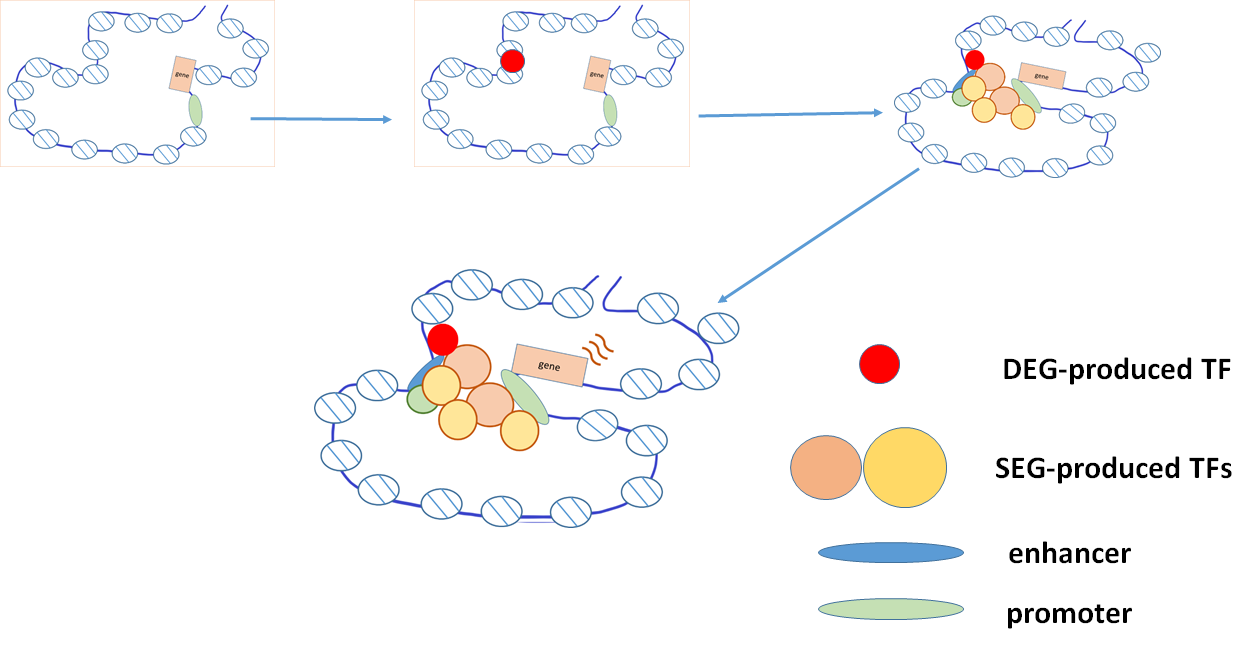


**Fig. S8.** Schematic representation of developmental enhancer- promoter activation with (a) binding DEG-produced TF to nucleosome (b) recruiting other TFs and transcription machinery (including SEG-produced TFs); (c) bridging corresponding target gene’s promoter by SEG-produced TFs, which all leads to the gene transcription.


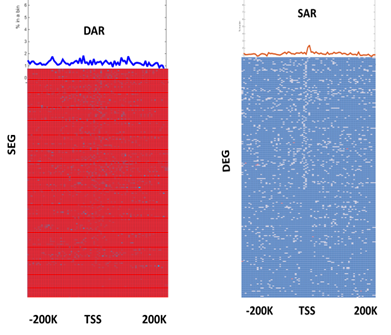


**Figure S9**. Heatmaps and profiles showing SARs co-localised with DEGs TSS and DARs co-localized with SEGs

**Supplementary Tables**

**Table S1.** Known developmental marker genes

| **gene name** | **known marker in lineage** | **reference** |
| --- | --- | --- |
| Crabp2, Irx3 | ectoderm | [^18^](https://sciwheel.com/work/citation?ids=7923921&pre=&suf=&sa=0) |
| Sox2, Nav2 | ectoderm | [**^118^**](https://sciwheel.com/work/citation?ids=6727215&pre=&suf=&sa=0) |
| Foxa2, Sox17 | endoderm | [**^61^**](https://sciwheel.com/work/citation?ids=1141295&pre=&suf=&sa=0) |
| Mesp1, Phida2, Lefty | mesoderm | [**^18^**](https://sciwheel.com/work/citation?ids=7923921&pre=&suf=&sa=0) |
| Gata4  Gata2 | mesoderm | **120**  **121** |

**Tables S2** GO MF Terms for DEGs and SEGs

**
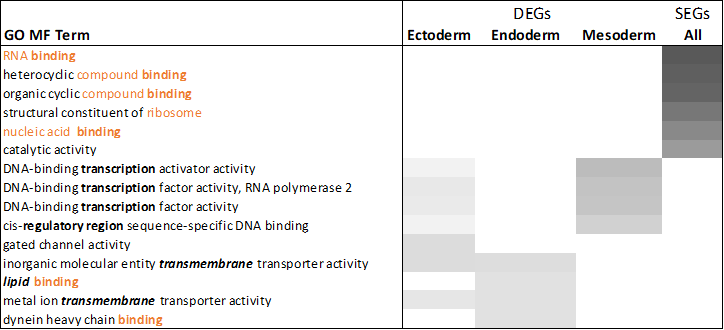
**

**Tables S3 extended. Specificity of enhancers-promoters between DEGs and SEGs regulation**

**Table S3.1 extended**: pairwise rmses between promoters

|  | prom Ect | prom End | prom Mes | prom SEG |
| --- | --- | --- | --- | --- |
| prom Ect | 0 | 0.06 | 0.075 | 0.1 |
| prom End |  | 0 | 0.05 | 0.11 |
| prom Mes |  |  | 0 | 0.105 |
| prom SEG |  |  |  | 0 |

**Table S3.2 extended** : pairwise rmses between enhancers

|  | enhancer Ect | enhancer End | enhancer Mes | enhancer SEG |
| --- | --- | --- | --- | --- |
| enhancer Ect | 0 | 0.066 | 0.08 | 0.12 |
| enhancer End |  | 0 | 0.077 | 0.11 |
| enhancer Mes |  |  | 0 | 0.1 |
| enhancer SEG |  |  |  | 0 |

Promoters DEGs are more similar to each other than to promoters SEG: t-test, p = 0.032

Enhancers DEGs are more similar to each other than to enhancers SEG: t-test, p = 0.012


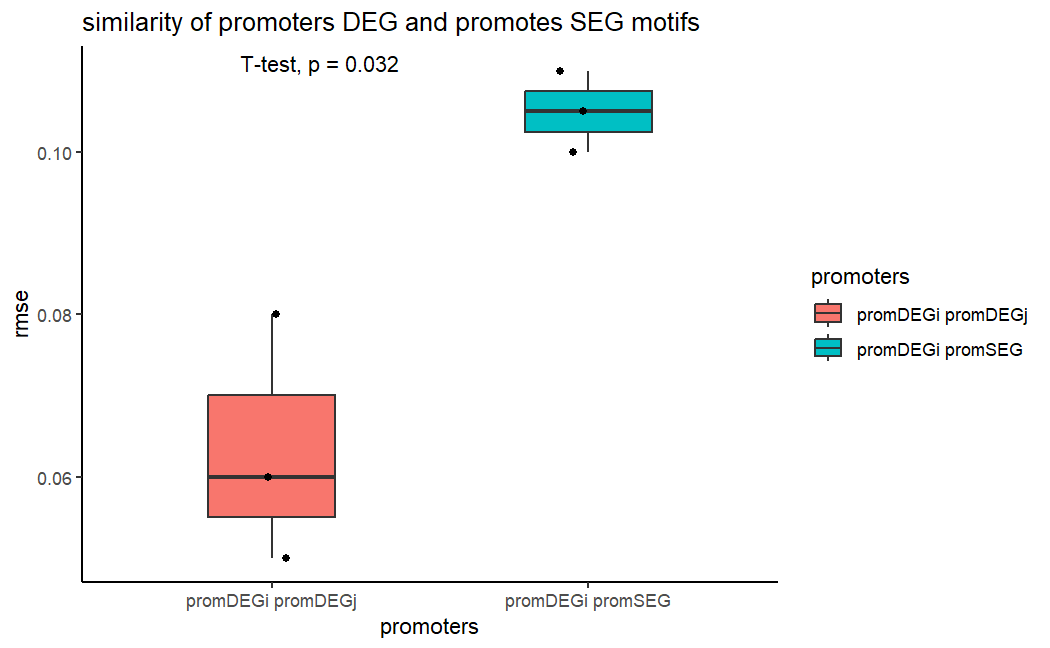

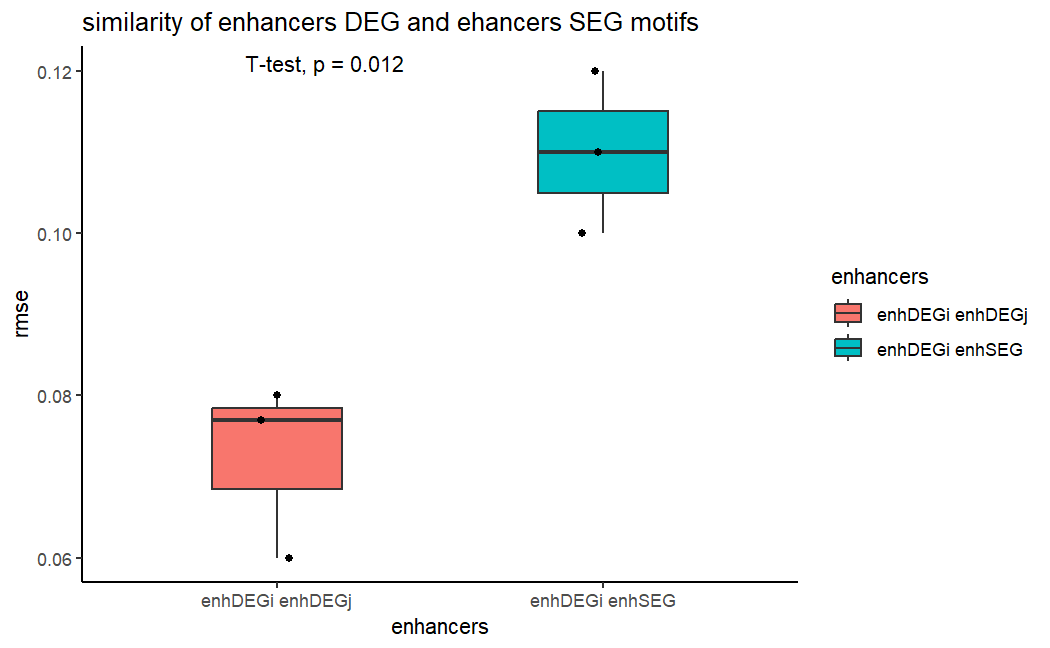


**Test S3.1-S3.2** **extended**.

(Left) T-test showing significant difference between promoters DEGs vs promoter SEG (left, median rmse scores are from Table S3.1). Promoters DEGs are more similar to each other than to promoters SEG: t-test, p = 0.032

(Right) T-test showing that enhancer DEG-enhancer SEG (right, median rmse scores are from TableS3.2) motif’s repertoire rmse/similarity. Enhancers DEGs are more similar to each other than to enhancers SEG: t-test, p = 0.012

**Table S3.3 extended**: pairwise rmses between enhancers and promoters

|  | enhancer Ect | enhancer End | enhancer Mes | enhancer SEG |
| --- | --- | --- | --- | --- |
| promoter Ect | 0.09 | 0.1 | 0.12 | 0.12 |
| promoter End | 0.12 | 0.11 | 0.1 | 0.14 |
| promoter Mes | 0.108 | 0.1 | 0.14 | 0.13 |
| promoter SEG | 0.126 | 0.145 | 0.13 | 0.11 |


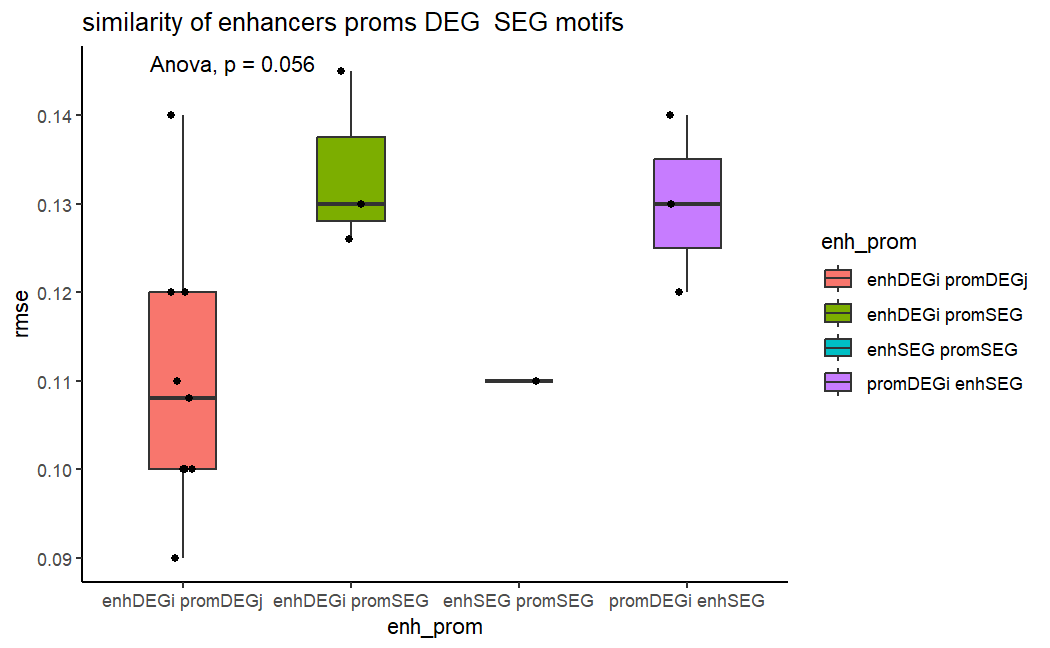


**Test S3.3** ANOVA test showing significant difference between ‘promoter DEG - enhancer DEG’ (orange), ‘promoter SEG- enhancer SEG’ (blue, one point) , ‘enhancer DEG-promoter SEG’ (green) and ‘enhancer DEG-enhancerSEG’ (violet) motif’s repertoire rmse/similarity. Median rmse values correspond to TableS3.3


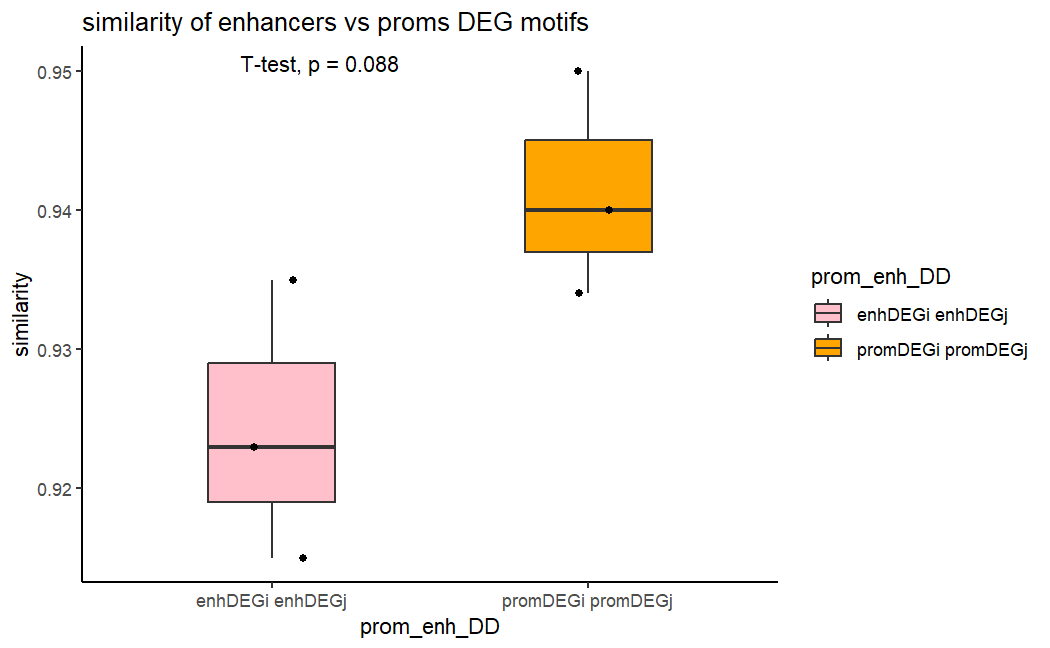


**Test S3.4**. T-test within DEGs showing that similarity (1-rmse) between DEG enhancers is lower than similarity between DEG’s promoters. It is significant at a=0.1.

**Table S4.** Pioneer and non-pioneer TF role, mentioned in a literature

| pioneer | Non-pioneer |
| --- | --- |
| **Ectoderm DEG-produced**  SOX2 (Zaret 2020)  (Larson 2021)  (Mayran and Drouin 2018)  POU3F1 (Swinstead 2016)  SOX3 (Swinstead 2016)  SOX2-OCT4 (Larson 2021) (Zaret 2020)  OCT4/POU5F1 (Larson 2021)  NANOG (Meers et al., 2019) | Ectoderm **SEG**  Zic2 (Larson 2021  ZIC3 (Larson 2021  ZFP281 (Fidalgo 2011)  MAZ (Chen 2020)  SOX4 (Chen 2020)  FOXN3 (Chen 2020) |
| **Endoderm DEG**  SOX17 (Cirillo 2002)  (Soufi 2015)  ( Zhu 2018)  (Meers 2019)  GATA1/3 (Meers 2019)  (Swinstead 2016)  FOXA1/2/3 (Cirillo 2002)  ( Iwafuchi-Doi 2016)  (Meers 2019)  EOMES (Meers 2019) | **Endoderm SEG**  ZIC3 (Larson 2021)  FOXP1 (Chen 2020)  FOXK2 (Chen 2020)**(Ji 2021)**  TRPS1 (Serandour 2018)  MAZ (Chen 2020)  RFX1/3/5 (Chen 2020)  SOX4 (Chen 2020) |
| **Mesoderm DE**G  TWIST2 ([Berkes et al. 2004](https://www.ncbi.nlm.nih.gov/pmc/articles/PMC1804332/#B8)) (Sandmann 2007)  LEF1 (Landry 2003) (Mok 2021)  GATA2 (Chaytor 2019) (Meers 2019)  MSGN1 (Chalamalasetty 2014)  TBX (Tapia-Carrillo 2019)  TCF12 (Landry 2003) (Mok 2021) | **Mesoderm SEG**  ZIC2 (Larson 2021)  ZIC3 (Larson 2021)  ZFP281 (Fidalgo 2011)  TCF3/4 (Chen 2020)  TRPS1 (Serandour 2018) |
