# Supplementary material for: Integrative analysis of transcriptomic and epigenomic data reveals distinct patterns for developmental and housekeeping gene regulation
Source: BMC Biol. 2024 Apr 10;22:78. doi: 10.1186/s12915-024-01869-2 (PMC11005181; doi:10.1186/s12915-024-01869-2)
Supplement: Supplementary file 5 — Additional file 5. TFBS motif names, common for all searched databases. [file 12915_2024_1869_MOESM5_ESM.docx]

AHR

AIRE

ALX1

ANDR

ARI5B

ARNT

ASCL1

ASCL2

ATF1

ATF2

ATF3

ATF4

ATOH1

BARX1

BATF3

BCL6

BHA15

BHE40

BMAL1

BRAC

CDX1

CDX2

CDX4

CLOCK

COT1

CREB1

CREM

CTCF

CTCFL

DBP

DLX5

DMRT1

DMRTB

E2F1

E2F3

E2F4

E2F6

E2F7

EGR1

EGR2

EHF

ELF1

ELF2

ELF3

ELF5

ELK1

ELK4

EPAS1

ERG

ERR1

ERR2

ERR3

ESR1

ESR2

ETS1

ETS2

ETV2

ETV4

ETV6

EVI1

EVX1

EVX2

FEV

FLI1

FOSB

FOXA1

FOXA2

FOXA3

FOXC1

FOXD3

FOXI1

FOXJ2

FOXJ3

FOXK1

FOXL2

FOXM1

FOXO1

FOXO3

FOXO4

FOXP2

FOXQ1

GABPA

GATA1

GATA2

GATA3

GATA4

GATA6

GCR

GFI1

GFI1B

GLI1

HAND1

HEN1

HIF1A

HLF

HNF1A

HNF1B

HNF4A

HNF4G

HSF1

HSF2

HTF4

HXA1

HXA10

HXA13

HXA9

HXB7

IKZF1

IRF1

IRF2

IRF3

IRF4

IRF7

IRF8

IRF9

ITF2

JUNB

KAISO

KLF1

KLF15

KLF3

KLF4

KLF5

KLF6

LEF1

LHX2

LHX3

LHX6

LYL1

MAFB

MAFK

MAX

MAZ

MECP2

MEF2A

MEF2C

MEF2D

MEIS1

MITF

MSGN1

MXI1

MYC

MYCN

MYF6

MYOD1

MYOG

NANOG

NDF1

NDF2

NF2L1

NFAC1

NFAC2

NFAC3

NFAC4

NFE2

NFIB

NFIL3

NFYA

NFYB

NFYC

NGN2

NKX61

NR1D1

NR1D2

NR1H3

NR2C1

NR2C2

NR2E3

NR4A1

NR4A2

NR5A2

NRF1

OLIG2

OTX2

OVOL1

P53

PBX1

PBX2

PBX3

PEBB

PIT1

PITX1

PKNX1

PO2F1

PO2F2

PO3F1

PO3F2

PO5F1

PPARG

PRD16

PRDM1

PRDM5

PRDM9

PRGR

PROP1

PRRX2

PTF1A

RARA

RARG

REL

REST

RFX1

RFX2

RFX3

RFX6

RUNX1

RUNX2

RUNX3

RXRA

RXRB

RXRG

SALL4

SIX2

SIX4

SMAD2

SMAD3

SMAD4

SMCA5

SNAI1

SNAI2

SOX10

SOX2

SOX3

SOX4

SOX5

SOX9

SP1

SP2

SP3

SP4

SP5

SP7

SPI1

SPIB

SRBP2

SRY

STA5A

STA5B

STAT1

STAT2

STAT3

STAT4

STAT6

STF1

SUH

TAF1

TAL1

TBP

TBX21

TCF7

TEAD1

TEAD2

TEAD4

TF7L1

TFE2

TFE3

TFEB

TGIF1

THA11

TWST1

TYY1

USF1

USF2

WT1

XBP1

ZBT17

ZEB1

ZFP42

ZFX

ZIC1

ZIC2

ZIC3

ZKSC1

ZN143

ZN281

ZN322

ZN335
